# Near-atomic architecture of Singapore grouper iridovirus and implications for giant virus assembly

**DOI:** 10.1038/s41467-023-37681-9

**Published:** 2023-04-12

**Authors:** Zhennan Zhao, Youhua Huang, Congcong Liu, Dongjie Zhu, Shuaixin Gao, Sheng Liu, Ruchao Peng, Ya Zhang, Xiaohong Huang, Jianxun Qi, Catherine C. L. Wong, Xinzheng Zhang, Peiyi Wang, Qiwei Qin, George F. Gao

**Affiliations:** 1 CAS Key Laboratory of Pathogen Microbiology and Immunology, Institute of Microbiology, Chinese Academy of Sciences, Beijing, 100101 China; 2 College of Marine Sciences, South China Agricultural University, Guangdong Laboratory for Lingnan Modern Agriculture, Guangzhou, 510642 China; 3 Cryo-EM Center, Department of Biology, Southern University of Science and Technology, Shenzhen, 518055 China; 4 Institute for Hepatology, National Clinical Research Center for Infectious Disease, Shenzhen Third People’s Hospital, Shenzhen, 518112 China; 5 School of Life Science, University of Science and Technology of China, Hefei, 230026 China; 6 National Laboratory of Biomacromolecules, Institute of Biophysics, Chinese Academy of Sciences, Beijing, 100101 China; 7 Clinical Research Institute, State Key Laboratory of Complex Severe and Rare Diseases, Peking Union Medical College Hospital, Chinese Academy of Medical Science & Peking Union Medical College, Beijing, 100730 China; 8 Department of Human Sciences & James Comprehensive Cancer Center, The Ohio State University, Columbus, 43210 USA; 9 Perelman School of Medicine, University of Pennsylvania, Philadelphia, 19104 USA; 10 University of Chinese Academy of Sciences, Beijing, 100049 China; 11 Beijing Life Science Academy, Beijing, 102209 China

**Keywords:** Cryoelectron microscopy, Virus structures, Pathogens

## Abstract

Singapore grouper iridovirus (SGIV), one of the nucleocytoviricota viruses (NCVs), is a highly pathogenic iridovirid. SGIV infection results in massive economic losses to the aquaculture industry and significantly threatens global biodiversity. In recent years, high morbidity and mortality in aquatic animals have been caused by iridovirid infections worldwide. Effective control and prevention strategies are urgently needed. Here, we present a near-atomic architecture of the SGIV capsid and identify eight types of capsid proteins. The viral inner membrane-integrated anchor protein colocalizes with the endoplasmic reticulum (ER), supporting the hypothesis that the biogenesis of the inner membrane is associated with the ER. Additionally, immunofluorescence assays indicate minor capsid proteins (mCPs) could form various building blocks with major capsid proteins (MCPs) before the formation of a viral factory (VF). These results expand our understanding of the capsid assembly of NCVs and provide more targets for vaccine and drug design to fight iridovirid infections.

## Abstract (web summary)

High morbidity and mortality in aquatic have been caused by iridovirids worldwide. Here the authors present a near-atomic SGIV capsid structure. Functional assays further reveal the relationships between identified capsid proteins and viral assembly.

## Introduction

Nucleocytoviricota viruses (NCVs) (https://talk.ictvonline.org/taxonomy), previously known as nucleocytoplasmic large DNA viruses (NCLDVs), are a diverse viral group infecting eukaryotes, all of which have a cytoplasmic phase and some also have a nuclear phase for viral replication^1^. Their giant double-stranded DNA genomes (up to 2.5 Mb) contain many genes involved in DNA replication, transcription, translation, and repair^2–5^, in contrast to most viruses with smaller genomes. Many NCVs, such as poxviruses, African swine fever virus (ASFV), and iridovirids, infect humans or important economic animals, threatening human health and causing enormous economic losses. Iridovirids, all members of the family *Iridoviridae*, are classified into two subfamilies (*Alphairidovirinae* and *Betairidovirinae*) and five genera (*Ranavirus*, *Megalocytivirus*, *Lymphocystivirus*, *Iridovirus*, and *Chloriridovirus*)^6^. The former three genera (subfamily *Alphairidovirinae*) infect ectothermic vertebrates, such as fish, amphibians and reptiles; the latter two (subfamily *Betairidovirinae*) infect invertebrate arthropods^6–8^. Infections by vertebrate iridovirids, including Singapore grouper iridovirus (SGIV), are associated with considerable morbidity and mortality in a wide range of wild and cultivated fish and amphibian species, resulting in massive economic losses and ecological destruction^9–18^. Effective control and prevention strategies to protect these animals are urgently needed.

The structural, physical, and biochemical characteristics of viral capsids are crucial for understanding the molecular mechanisms that control successful capsid assembly^19^. Determining viral structures at high resolution and exploring the capsid assembly process could provide pivotal information for identifying structure-function relationships^20–22^. Most NCVs have an icosahedral capsid, except poxviruses^23^, ascoviruses^24^, pandoraviruses^25^, pithoviruses^26^, and molliviruses^27,28^. Previous studies report the cryogenic electron microscopy (cryo-EM) structures of ASFV^29–31^, SGIV^32^, Chilo iridescent virus (CIV)^33,34^, Paramecium bursaria chlorella virus 1 (PBCV-1)^33,35–37^, Acanthamoeba polyphaga mimivirus (APMV)^38^, Phaeocystis pouchetti virus (PpV01)^39^, Melbourne virus^40^, Cafeteria roenbergensis virus (CroV)^41^, faustovirus^42^, and pacmanvirus^43^. However, the high-resolution structure determination of these viral capsids is greatly hindered by the Ewald sphere effect, the large size and the intrinsic flexibility of the capsid components^44–46^. To date, only the structures of the PBCV-1^37^ and ASFV^29,30^ capsids have been resolved to near-atomic resolution.

Here, we report the cryo-EM structure of the SGIV capsid at 3.5 Å resolution using a block-based reconstruction method^44^. The cryo-EM map, combined with viral proteomics, crosslinking mass spectrometry (CXMS) and fluorescence colocalization assays, enables us to identify the major capsid protein (MCP) and seven types of minor capsid proteins (mCPs). The atomic models for an asymmetric unit (ASU) of the viral capsid constituted by different copies of these capsid proteins are further determined. In addition, fluorescence colocalization assays show that the anchor protein integrated with the viral inner membrane colocalizes with the endoplasmic reticulum (ER), suggesting an association between the viral inner membrane and the ER membrane. The observation that mCPs colocalize with the MCP in the cytoplasm of SGIV-infected cells before viral factory (VF) occurrence indicates that the virus could use diverse building blocks formed by mCP and MCP complexes for capsid assembly. These findings expand our understanding of the capsid assembly of the icosahedral NCVs and provide new clues for developing drugs and vaccines to fight iridovirid infections.

## Results

### Overall architecture of the SGIV capsid

**Fig. 1:**
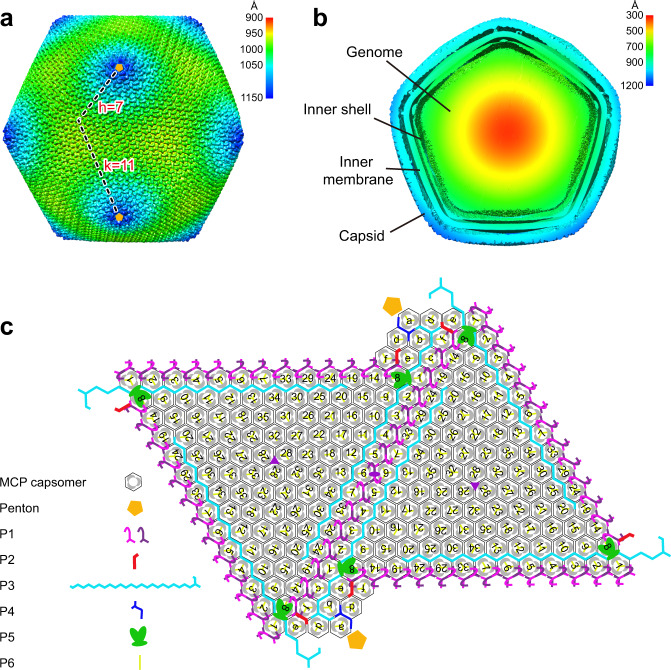
Overall architecture of SGIV capsid. **a** Icosahedrally averaged cryo-EM reconstruction of the viral capsid, which is colored by the radial distance from the virus center. h and k vectors constituting the T number are shown as dashed lines. The pentameric capsomers (penton) are presented as solid orange pentagons, and each penton protein occupies a vertex of a pentagon. **b** Density map slice showing multiple layers of the SGIV virion, which is colored by the radial distance from the virus center. **c** Diagrammatic organization of the MCP trimer and mCPs viewed inside the virion. MCP capsomers are outlined as hexagons, in which each gray lune represents a subunit. The mCPs (penton and P1-P6) are shown as different shapes with different colors, as indicated. Notably, two of the same shapes with different colors are used to represent two P1 protomers, which are interlocked into one homodimer in the zip band. MCP capsomers within trisymmetrons are labeled in numeric order (1, 2, 3…; 35 in total in one ASU), and those within pentasymmetrons are labeled in alphabetic order (a, b, c, d, e, and f; six in total in one ASU).

The reconstructed viral capsid has a diameter of ~228 nm. Capsomers on the capsid are arranged in an icosahedral fashion, where *h* = 7 and *k* = 11 give a triangulation (T) number of 247 (Fig. 1a). Like ASFV^29,30,47^, SGIV particles are composed of icosahedral multilayered structures, consistent with a previous study^32^. Due to the SGIV sample being purified by three cycles of freezing and thawing the mixture of the cells and supernatant, followed by detergent treatment, four multilayered structures including a genome, inner shell, inner membrane, and outer capsid are visible inside to outside without an outer membrane observed (Fig. 1b). In addition, as observed in many NCVs^30,34,37,39,41,42^, SGIV capsomers are organized into 12 pentasymmetrons and 20 trisymmetrons (Supplementary Fig. 1). The pentasymmetron is centered on the fivefold vertex, consisting of 30 MCP capsomers (MCP trimers), and one pentameric capsomer (penton) (Fig. 1c and Supplementary Fig. 1). The trisymmetron comprises 105 MCP capsomers and is centered on the icosahedral threefold axis (Fig. 1c and Supplementary Fig. 1). MCP capsomers display a rotation of 60° between two neighboring trisymmetrons, resulting in the formation of icosahedral edges (Fig. 1c and Supplementary Fig. 1).

Previous work shows that multiple types of mCPs form a network underneath the pentons and MCPs, among which many types of mCPs have been defined, such as anchor protein, zip protein, finger protein, and tape-measure protein (TmP)^29,30,34,37^. However, the types and stoichiometries of mCPs in these NCVs are distinct. For instance, the anchor protein and finger protein are reported in CIV^34^ but not in ASFV^29,30^ and PBCV-1^37^; TmP is observed in the capsids of ASFV and PBCV-1 but not in CIV. Additionally, 14 different types of mCPs have been identified in the PBCV-1 capsid^37^, significantly more than that of ASFV^29,30^ and CIV^34^. Notably, only atomic models of PBCV-1 mCPs are available^37^. Because the protein sequences of the mCPs among NCVs share little similarities, determining the protein sequences or the coding genes of mCPs in other NCVs is still challenging.

To visualize the mCPs of SGIV, MCP densities were subtracted from the overall density map. Seven different types of mCPs (penton protein and P1-P6) were identified (Fig. 1c). These mCPs are positioned at the interfaces between neighboring MCP capsomers, except for the penton protein that forms a pentamer (penton) in each fivefold vertex and P6, which fills the inner cavity of each MCP capsomer (Fig. 1c). In one ASU, there are 41 MCP capsomers, of which six are in the pentasymmetron, and the other 35 are in the trisymmetron (Fig. 1c). Moreover, one copy of the penton protein, P2, P3, P4, and P5, 15 copies of P1, and 123 copies of P6 were observed in one ASU (Fig. 1c).

### Structure and organization of the MCP

**Fig. 2:**
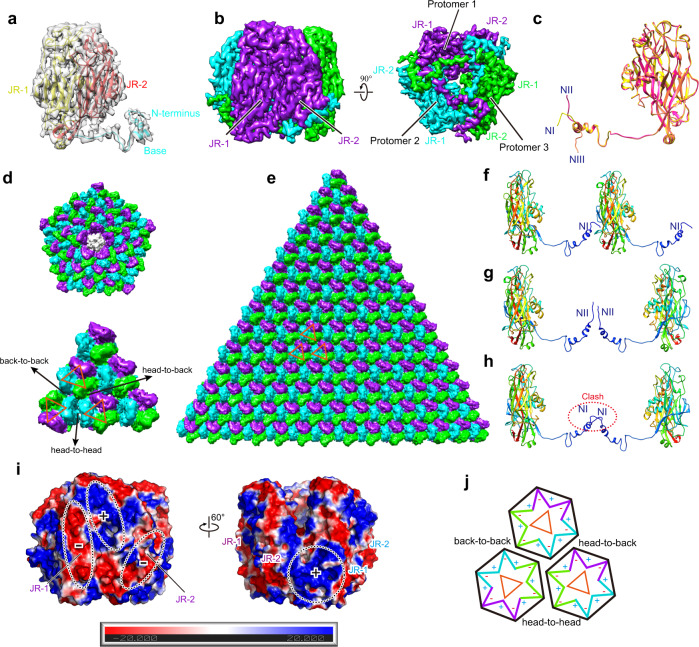
Structure and arrangement of the SGIV MCP. **a** Atomic model and density map of one VP72 monomer. The double JR domains (JR-1 and JR-2) are colored in yellow and red, respectively. Additionally, the N-terminal base is colored in cyan, and the C-terminus is colored in orange. **b** Cryo-EM map of one trimeric VP72 capsomer, three protomers of which are colored in purple, cyan, and green. Six JR domains within one trimer form the vertices of a pseudo-hexamer. Each N-terminal base extends along the bottom of the neighboring protomer until the interface formed by another two protomers. **c** Three conformations of N-terminal residues of the MCP protomers, labeled NI (yellow), NII (hot pink), and NIII (orange). **d**, **e** The arrangement of MCP capsomers in symmetrons. The groove formed by JR-1 and JR-2 in one protomer is shown as the vertex of a triangle (orange) that is used to represent the orientation of the MCP capsomer. Each vertex of the triangle is named a head, and the edge is called a back. In the pentasymmetron, there are three different arrangements: head-to-head, head-to-back, and back-to-back (**d**). In the trisymmetron, only the head-to-back arrangement is observed (**e**). **f** The N-terminal conformation of two adjacent MCPs in the head-to-back arrangement. **g** The N-terminal conformation of two adjacent MCPs in the back-to-back arrangement. **h** Simulation of a back-to-back arrangement using the MCPs in the head-to-back arrangement. The clash region is boxed in the red ellipse. **i** Electrostatic potential distribution of the MCP capsomer. Two JR domains (JR-1 and JR-2) are dominated by negative charges, but the groove between two JR domains in one protomer and the interface between two neighboring protomers are mainly positively charged. **j** Schematic diagram of three different arrangements of MCP capsomers in SGIV.

The MCP is the most abundant virion component and is named VP72 because it is encoded by the *orf72* gene of the SGIV genome. Similar to the MCPs of many NCVs^42,48–50^, bacteriophages^51–53^, virophages^54,55^, adenoviruses (AdV)^53,56,57^, and *Sulfolobus* turreted icosahedral virus (STIV)^58^, the trunk region of VP72 displays a typical double jelly roll (JR) fold (JR-1 and JR-2) (Fig. 2a and Supplementary Fig. 2); each JR fold comprises two β-sheets formed by eight antiparallel β-strands^57,59^ (Supplementary Fig. 2). Three VP72 protomers trimerize to form the MCP trimer, named the pseudo-hexameric capsomer due to its hexagonal shape^60,61^ (Fig. 2b). The N-terminal base of the VP72 protomer (Fig. 2a) extends along the bottom of the adjacent protomer until the interface formed by the other two protomers in one capsomer (Fig. 2b).

**Fig. 3:**
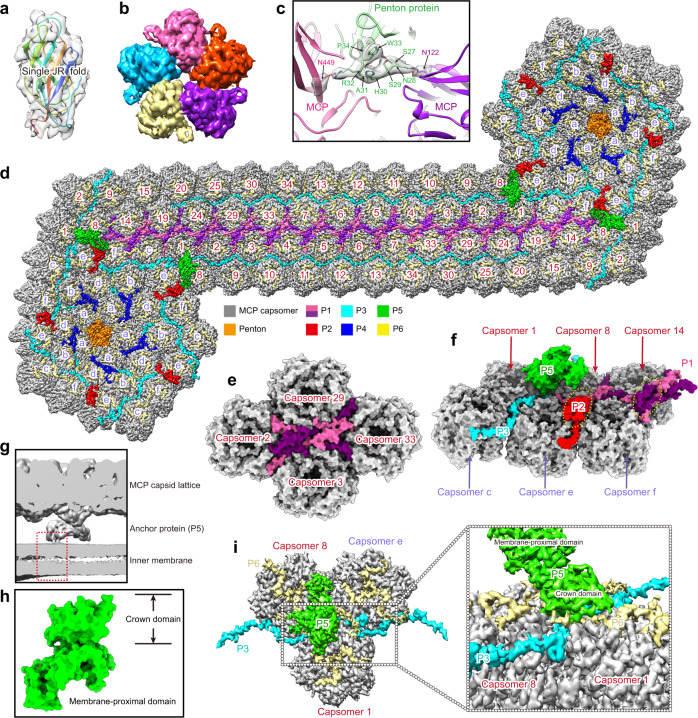
Structures and distributions of mCPs. **a** Atomic model and density map of one penton (VP14) monomer. Rainbow coloring from blue to red indicates the N- to C-terminus of the residues. **b** A pentamer formed by five penton proteins in the fivefold vertex. Five penton proteins are colored in khaki, purple, orange red, hot pink, and deep sky blue, respectively. **c** Each penton protein contacts two adjacent MCP protomers. The penton protein is colored green, and two MCP protomers are colored hot pink and purple. The interface is viewed inside the virion. H-bonds are labeled as red dashed lines, and corresponding residues are shown as sticks. The local density map with the model fitted covering the above contact residues is shown as gray surfaces. **d** Overall arrangement of mCPs beneath the zipper region and pentasymmetron, which is viewed inside the virions. One zipper band links two adjacent pentasymmetrons together. MCPs and mCPs are colored corresponding to Fig. 1c, and the MCP capsomers are numbered as in Fig. 1c. **e** The atomic models of one P1 (zip protein) dimer and its neighboring MCP capsomers. Two protomers of the P1 dimer are shown as hot pink and purple surfaces. Its N-terminal sequence, which was predicted to be associated with the membrane, is invisible in the cryo-EM map due to its flexibility. MCP capsomers surrounding the P1 dimer are shown as gray surfaces and numbered corresponding to (**d**). **f** An incomplete P1 dimer at the boundary between the zipper band and pentasymmetron. Another mCP (P2) occupies the space generated by the loss of AA of the zip protein. The atomic models of MCPs, P1, P2, P3 and P5 are shown as surfaces and colored corresponding to (**d**). Additionally, MCP capsomers are numbered and labeled, as shown in (**d**). **g** Anchor protein (P5) is anchored to the viral inner membrane. P5, the MCP capsid lattice, and the inner membrane are labeled. **h** The ectodomain of the anchor protein, colored corresponding to (**d**), can be divided into a crown domain and a membrane-proximal domain. **i** Intermolecular contacts between the anchor protein and its neighboring capsid proteins. The density maps of the capsid proteins are shown and colored corresponding to (**d**).

The MCPs of PBCV-1 have three significantly different conformations in their N-terminal residues^37^. To explore the conformational differences among SGIV MCPs, the 123 MCP protomers in one ASU were structurally aligned. Similar to PBCV-1^37^, the structural alignment analysis revealed that the N-terminal residues of the SGIV MCPs are conformationally diverse and can be grouped into three configurations (NI, NII and NIII). NI displays a hook-like shape, NII is perpendicular to the capsid surface, and NIII is parallel to the bottom of the capsid (Fig. 2c). As described in PBCV-1^62^ and ASFV^29,30^, three arrangements (head-to-back, head-to-head, and back-to-back) were observed in the pentasymmetron (Fig. 2d). In the trisymmetron, all capsomers adopt the head-to-back arrangement (Fig. 2e). The MCPs with a head-to-back arrangement adopt the NI conformation (Fig. 2f). Most MCPs with back-to-back arrangement adopt the NII conformation (Fig. 3g). Notably, like that mentioned in PBCV-1^37^, the MCP capsomer c, which has a back-to-back arrangement with capsomer b, adopts the NIII configuration in its N-terminal residues and closely contacts P3. Furthermore, the N-terminal residues with the NI conformation of the MCPs adjacent to mCPs also display slight conformational changes. The MCPs with the NI conformation were further used to simulate the back-to-back arrangement, which indicates a severe clash in their N-terminus (Fig. 2h). These observations suggest that conformational adjustment for the N-terminus of MCPs could be crucial for forming and stabilizing the capsid lattice, and mCPs could have an impact on the N-terminal conformation of MCPs.

Previous work suggests that electrostatic interactions play a vital role in the capsid assembly of PBCV-1^62^ and ASFV^29^. The electrostatic potential distribution of the SGIV MCP was analyzed, which shows that both JR-1 and JR-2 are dominated by negative charges (Fig. 2i). In contrast, the groove formed by JR-1 and JR-2 in the same MCP protomer is mainly positively charged, as well as the interface between JR-1 and JR-2 of two neighboring MCP protomers (Fig. 2i). In the MCP lattice, the neighboring MCP capsomers are arranged in a slightly dislocated manner, which results in a JR domain of one MCP capsomer being inserted into the groove or interface between two adjacent JR domains of the other MCP capsomer (Fig. 2d, e, j). The alternating distribution of positive and negative charges of the SGIV MCP may help stabilize the capsomer lattice by charge complementarity in all three arrangements (Fig. 2j).

### Structures and distributions of mCPs

We attempted to search for the protein sequence of the SGIV penton protein from 162 predicted open reading frames (ORFs)^63^ by amino acid (AA) sequence alignment to penton proteins with a known structure^37,54,55,64,65^. However, no genes with significant similarities were found. Based on the information from the cryo-EM map and proteomics of the purified SGIV sample (Supplementary Table 1), the penton protein was ultimately determined to correspond to the VP14 protein, which is a polypeptide encoded by the *orf14* gene and harbors eight potential β-strands (Supplementary Fig. 3). Like other penton proteins (Supplementary Fig. 4)^37,54,55,64,65^, the atomic model of VP14 displays a single JR fold with no apparent top crown (Fig. 3a). Five VP14 protomers form a pentameric capsomer (penton) (Fig. 3b). Each VP14 protomer contacts two MCP protomers from two adjacent pseudo-hexameric capsomers (Fig. 3c). Residues N28 and R32 of the penton protein are hydrogen-bonded with residue N122 from one MCP protomer and N449 from the other MCP protomer, respectively (Fig. 3c).

Similar to PBCV-1^37^, CIV^34^, FAUV^42^, and ASFV^29,30^, a zipper band, formed by MCP capsomers and various mCPs, glues adjacent trisymmetrons together and links neighboring pentasymmetrons. Furthermore, a zip protein (P1) array was also observed in the zipper band (Fig. 3d), which is thought to help stabilize the capsid shell. However, the AA sequences and structures of these zip proteins have not been identified except for that of PBCV-1^37^. Using the method described above for the SGIV penton protein, the SGIV zip protein was identified as the VP38 protein encoded by the *orf38* gene. Secondary structure prediction indicates that VP38 might be a single transmembrane protein (Supplementary Fig. 5). Given that the density map of VP38 is located between the viral inner membrane and the outer capsid shell, its transmembrane region could be associated with the inner membrane. However, its transmembrane region is invisible in the density map, possibly due to its flexibility. The atomic model of VP38 (P1) shows that two VP38 protomers are interlocked into a dimer (Fig. 3e), which may contribute to gluing two antiparallel MCP capsomers together. Notably, an incomplete VP38 (P1) dimer in the interface formed by capsomer 8, 14, e, and f is observed (Fig. 3f). Compared with other VP38 protomers, residues 77–87 and 112–124 in one protomer of this dimer and residues 129–149 in the other are missing. The space generated by these missing residues is occupied by another mCP (P2) VP139 (Fig. 3f and Supplementary Fig. 6). The reason why this VP38 dimer is incomplete and VP139 is recruited here is unknown.

As observed in ASFV^29,30^, PBCV-1^37^, and bacteriophage PRD1^64,66^, an unstructured filament-like structure named TmP (P3) was also found in SGIV (Fig. 3d), which is thought to regulate the size and shape of the virus^61,67^. The TmP (P3) of SGIV corresponds to the VP137 protein encoded by the *orf137* gene (Supplementary Fig. 7). Two antiparallel TmPs wind along two sides of the zipper band to connect neighboring pentasymmetrons (Fig. 3d). Close to the N-terminus of TmP is another mCP (P4), VP59 (Supplementary Fig. 8), which fills the gaps formed by the capsomer, a, b, and d (Fig. 3d).

Anchor protein was previously reported in CIV^34^. However, its sequence and atomic structure remain to be determined. VP88, encoded by the *orf88* gene, was identified as the anchor protein (P5) of SGIV. The cryo-EM map shows that its prominent transmembrane regions are inserted into the viral inner membrane (Fig. 3g), consistent with the secondary structure prediction (Supplementary Fig. 9). The areas of VP88 outside the inner membrane could be divided into a top crown domain and an α-helical-abundant membrane-proximal domain (Fig. 3h). The membrane-proximal domain is anchored to the viral inner membrane, and the crown domain contacts MCPs (capsomer 1 and 8) and P6 (Fig. 3i). Notably, the densities of the capsid proteins mentioned above are better than that of VP88, and the density of VP88’s crown domain is better than that of its membrane-proximal domain, which may be caused by VP88’s proximity to the inner membrane. Many studies demonstrate that N-terminally myristylated proteins play an essential role in viral internal membrane biogenesis and virion assemblies, such as several myristylated proteins (L1R, A17L, and A14L) of vaccinia virus^68,69^, the Gag proteins of most retroviruses^70–73^, and picornavirus capsid protein VP4^74^. Thirteen iridovirids’ genomes covering five genera of the family *Iridoviridae* were searched for VP88 homologs using the basic local alignment search tool (BLAST)^75,76^, and the AA sequence alignment of the anchor protein in these iridovirids was generated by ClustalW and ESPript 3.0^77^. A typical myristylation site (S-G-X-X-X-S/T/A)^78^ was observed in the N-terminus of these anchor proteins (Supplementary Fig. 10), suggesting that they may be associated with viral internal membrane biogenesis and viral assembly.

In ASFV and AdV, additional densities are observed in the inner cavity of the MCP capsomer or hexon; they can be divided into three parts, which are thought to be three copies of one mCP^29,30^ or three different mCPs^56^. In AdV, these proteins play critical roles in genome condensation^79^ and capsid disassembly^80,81^. In the cryo-EM map of SGIV, additional densities were also observed to fill the internal cavity of all MCP capsomers in pentasymmetrons and trisymmetrons (Figs. 1c, and 3d). Like ASFV, these three parts of SGIV have the same structural features in the density map, suggesting that they are three copies of one mCP (P6).

Due to its short visible polypeptide length (~40 AA) in the cryo-EM map, identifying the protein sequence of P6 based on information from the density map and the viral proteomics is difficult. To determine this protein from the 162 genes of the SGIV genome, CXMS analysis of the purified SGIV sample was further performed. We found that residue K47 of VP22 crosslinks to residue K57 of VP38 (P1) (Supplementary Fig. 11), indicating that VP22 is a structural protein of SGIV virions. Additionally, the density map shows that VP38 (P1) is close to P6 in the zipper band (Fig. 3d), which enables crosslinking. VP22 is also detected in the proteomics profile of the purified SGIV sample (Supplementary Table 1), supporting that it is a component of the SGIV virion. Altogether, these results indicate that VP22 is a strong candidate for P6. According to its secondary structure prediction, VP22 might be highly flexible and lack prominent secondary structure elements (Supplementary Fig. 12), which could be the reason why only a small patch is visible in the density map.

### Colocalization between mCPs and MCP

**Fig. 4:**
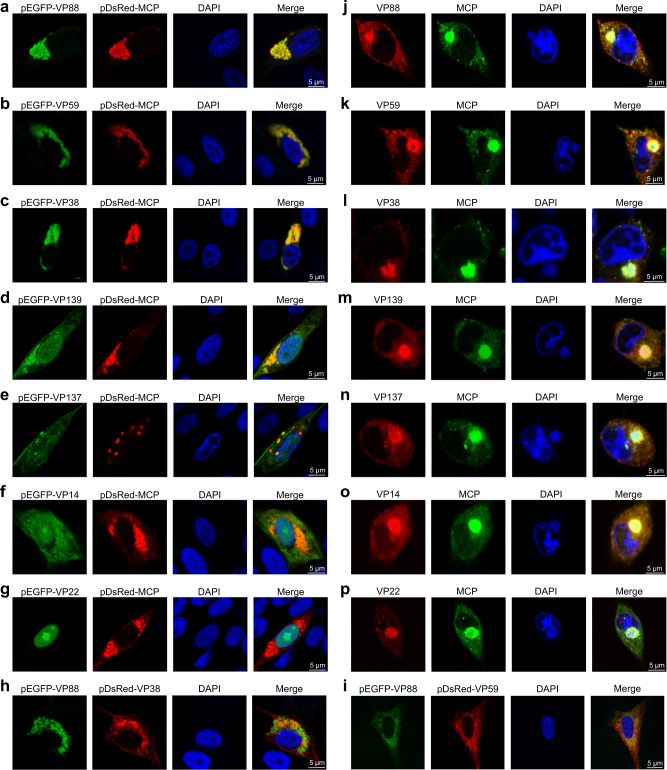
Colocalization between mCPs and MCP. Fluorescence colocalization assays between the mCP including VP88 (**a**), VP59 (**b**), VP38 (**c**), VP139 (**d**), VP137 (**e**), VP14 (**f**), or VP22(**g**) and the MCP after cotransfection with the recombinant plasmid (pEGFP-VP88, pEGFP-VP59, pEGFP-VP38, pEGFP-VP139, pEGFP-VP137, pEGFP-VP14 or pEGFP-VP22) expressing the mCP fused to EGFP and the pDsRed-MCP plasmid expressing the MCP fused to DsRed into EAGS cells. Colocalization analysis between VP88 and VP38 (**h**), or between VP88 and VP59 (**i**) was performed as negative controls. Colocalization analysis between mCP including VP88 (**j**), VP59 (**k**), VP38 (**l**), VP139 (**m**), VP137 (**n**), VP14 (**o**), or VP22(**p**) and the MCP determined by immunofluorescence assays for the EAGS cells infected with authentic SGIV for 24 h. After fixation and permeabilization, cells were incubated with a mouse mAb against SGIV MCP and rabbit polyclonal antibody against SGIV VP88 (VP59, VP38, VP139, VP137, VP14, or VP22) for 1 h. Anti-rabbit Alexa Fluor 555 (red) secondary antibody and anti-mouse Alexa Fluor 488 (green) secondary antibody were used. The VF, a spherical structure close to the nucleus, was stained with DAPI. Data are representative of three independent experiments.

To validate whether the protein sequences of these mCPs were identified correctly, fluorescence colocalization assays between mCPs and the MCP in cotransfected cells were performed. After cotransfection of one mCP (penton protein or P1-P6) with the MCP, fluorescent signals from the mCP (green), MCP (red) and nucleus (blue) were detected. We found that VP88 (Fig. 4a), VP59 (Fig. 4b), or VP38 (Fig. 4c) distribute throughout the cytoplasm as well as the MCP, and they colocalized with the MCP in the cytoplasm. VP139 (Fig. 4d), VP137 (Fig. 4e), or VP14 (Fig. 4f) distributed in the cytoplasm and nucleus, in contrast to the MCP that was in the cytoplasm. The proteins (VP139, VP137, or VP14) in the cytoplasm partially colocalized with the MCP (Fig. 4d–f). However, in the cells cotransfected with VP22 and MCP, VP22 was completely localized in the nucleus and did not colocalize with the MCP still in the cytoplasm (Fig. 4g). VP88 (P5) and VP38 (P1) were not observed to contact each other in the cryo-EM density map nor VP88 (P5) and VP59 (P4) (Fig. 3d, f). Fluorescence colocalization assays between VP88 (P5) and VP38 (P1) and between VP88 (P5) and VP59 (P4) were thus performed as negative controls. We found that VP88 did not colocalize with VP38 (Fig. 4h) or VP59 (Fig. 4i) in the cotransfected cells.

Immunofluorescence assays were also performed at 24 h post infection (h p.i.) in Red-spotted grouper (*Epinephelus akaara*) spleen (EAGS) cells infected with authentic SGIV. At that time, the VF, where viral components concentrate^82^ for viral genome replication and virion assembly^47,83–89^, was observed as a spherical perinuclear or cytoplasmic structure and could be stained by DAPI (Sigma). The immunofluorescence assays demonstrated that all seven mCPs, including VP22, colocalized with the MCP in the VF (Fig. 4j–p), indicating that the seven identified mCPs participate in virion assembly like the MCP.

### ER localization of the anchor protein VP88

The assembly of ASFV at the VF is proposed to commence with the characteristic open curved membrane structures, which are thought to be collapsed cisternal domains recruited from the surrounding ER^90–92^. A previous study indicates that p54 of ASFV, an ER-targeted membrane protein, plays a critical role in the recruitment and transformation of ER membranes to form precursors of the inner membrane of virions^93^. Repression of ASFV p54 arrests virus morphogenesis at a very early stage, even prior to the formation of the precursor membranes, accompanied by the VF being essentially free of viral structures^93^. Scanning transmission electron microscopy tomography analyses of PBCV-1-infected cells also show that the precursors of the inner membrane appear to bud out from rough ER membranes^84^. The origin of the Mimivirus inner membrane is also suggested to be derived from the host ER network^94^. However, the viral proteins of PBCV-1 and Mimivirus that function like ASFV p54 remain to be identified.

Cryo-EM and cryo-electron tomography imaging of SGIV-infected cells show that membranous structures in the VF emerge as precursors to recruit capsid proteins to form an intermediately crescent-shaped structure, which curves to form icosahedral capsids^89^. The 53 R protein of the Rana grylio virus (RGV), a ranavirus belonging to the family *Iridoviridae*, initially colocalizes with ER components at an early stage post-transfection^78^. Another study shows that knocking down the expression of the Frog virus 3 (FV3) ORF53R gene, a homolog of RGV 53 R, blocks the formation of viral membrane precursors and whole virions in the VF^95^. All of these results support the notion that the precursors of the inner membrane for commencing the capsid assembly of NCVs may derive from the ER network. The 53R proteins of RGV and FV3 could be essential for the recruitment and transformation of ER membranes to form precursors of the inner membrane, like p54 of ASFV.

**Fig. 5:**
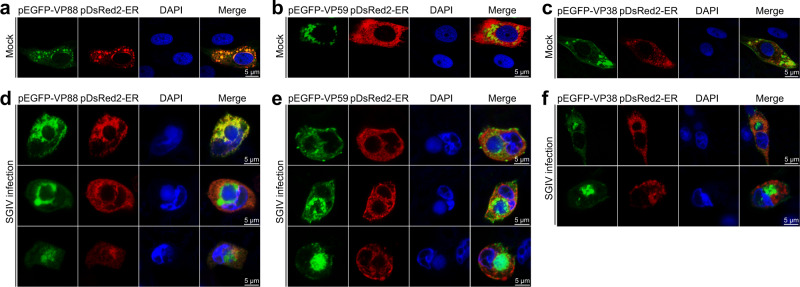
ER localization of the anchor protein. Localization of VP88 (**a**), VP59 (**b**), or VP38 (**c**) with DsRed2-ER (an ER marker) after cotransfection with the plasmid (pEGFP-VP88, pEGFP-VP59, or pEGFP-VP38) expressing one mCP fused to EGFP and pDsRed2-ER plasmid into EAGS cells. Multiple localization states between VP88 (**d**), VP59 (**e**), or VP38 (**f**) and DsRed2-ER observed in the EAGS cells that were cotransfected with pEGFP-VP88 (pEGFP-VP59 or pEGFP-VP38) and pDsRed2-ER, then infected with SGIV at MOI of 10. Results are representative of three independent experiments.

The VP88 protein (anchor protein of SGIV) shares a 57.81% (or 58.01%) AA sequence identity with RGV 53R (or FV3 53R). Furthermore, the transmembrane regions of VP88 are located in the viral inner membrane of SGIV (Fig. 3g). These findings indicate that VP88 could have the same functions as 53R of RGV or FV3. The transmembrane region prediction using Phyre2^96^ and TMHMM^97,98^ indicated that three types of mCPs (VP38, VP59, and VP88) have potential membrane-associated functions. To explore the association of these three mCPs with the ER, we examined whether these three mCPs are positioned in the ER. After cotransfection of VP38, VP59, or VP88 with DsRed2-ER (an ER marker) into EAGS cells, VP88 was observed to colocalize with the ER (Fig. 5a). However, the other two predicted membrane proteins, VP59 (Fig. 5b) and VP38 (Fig. 5c), both of which distributed in the cytoplasm, did not colocalize with the ER.

After incubation with SGIV following the cotransfection, multiple colocalization states between VP88 and the ER were observed. In some cotransfected SIGV-infected cells, VP88 was located on the ER (Fig. 5d), as observed in the cotransfected non-SGIV-infected cells (Fig. 5a). In some other cells, VP88 aggregated around the VF accompanied by its detachment from the ER (Fig. 5d). In addition, there were some cells in which the majority of VP88 colocalized with the VF, and ER aggregated proximally to the VF (Fig. 5d). These states may reflect a process in which VP88 is initially located at the ER and is then recruited from the ER to the VF and participates in the viral assembly in the VF. Notably, VP88 was separate from the ER during the migration toward the VF, which was previously observed in RGV-infected and 53R-DsRed2-ER-cotransfected cells and supports the notion that the process of recruitment appears to modify or damage the cellular ER so that proteins or other components in the ER (e.g., the ER marker) are prohibited from entering the VF^78,90^. After SGIV infection, VP59 was observed to aggregate toward the VF in some VP59-DsRed2-ER-cotransfected cells and colocalize with the VF in other cells (Fig. 5e). Similar results were also observed in the VP38-DsRed2-ER-cotransfected SGIV-infected cells (Fig. 5f).

Altogether, these findings indicate that the viral inner membrane-anchored VP88 is an ER-integrated membrane protein, which may function in the recruitment and transformation of ER membranes to form precursors of the viral inner membrane. However, the membrane types targeted by VP59 and VP38 remain to be further elucidated.

### Colocalization between mCPs and the MCP before the formation of the VF

**Fig. 6:**
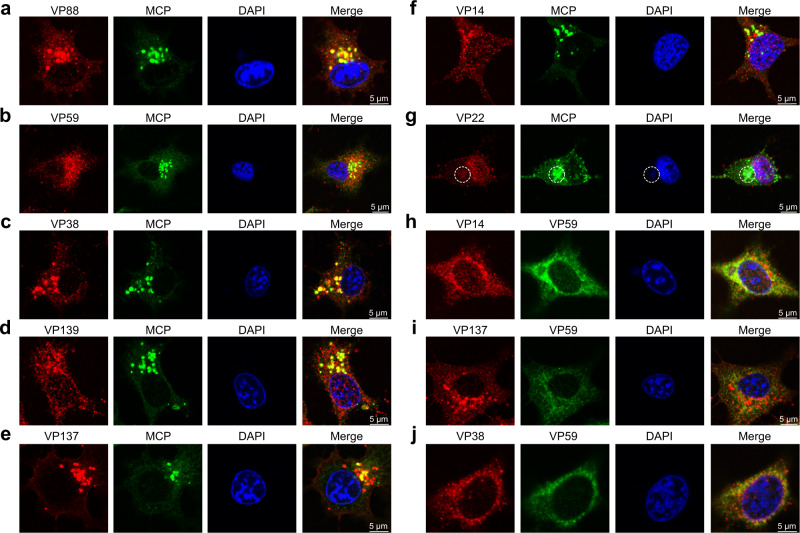
Colocalization between mCPs and MCP before the formation of the VF. Immunofluorescence colocalization assays between the mCP including VP88 (**a**), VP59 (**b**), VP38 (**c**), VP139 (**d**), VP137 (**e**), VP14 (**f**), or VP22(**g**) and the MCP in the SGIV-infected EAGS cells before the formation of the VF (12 h p.i.). After fixation and permeabilization, cells were incubated with a mouse mAb against SGIV MCP and rabbit polyclonal antibody against SGIV VP88 (VP59, VP38, VP139, VP137, VP14, or VP22) for 1 h. Anti-rabbit Alexa Fluor 555 (red) secondary antibody and anti-mouse Alexa Fluor 488 (green) secondary antibody were used. The area of colocalization between VP22 and the MCP in the cytoplasm is highlighted in a white dotted circle (**g**). Immunofluorescence colocalization assays between VP59 and VP14 (**h**), between VP59 and VP137 (**i**), or between VP59 and VP38 (**j**) were also performed. After fixation and permeabilization, cells were incubated with mouse polyclonal antibody against VP59 and rabbit polyclonal antibody against VP14 (VP137 or VP38), and the corresponding secondary antibodies described above were used. Finally, cells were stained with DAPI and observed by confocal laser scanning microscopy. One representative experiment out of three is shown.

To identify the colocalization patterns of various capsid proteins during authentic virus infection, we both examined their intracellular distributions after the formation of the VF (Fig. 4j–p) and determined their colocalization before the occurrence of the VF using immunofluorescence staining (Fig. 6). At the early stage of SGIV infection (12 h p.i., VF not formed), VP88 (Fig. 6a), VP59 (Fig. 6b), VP38 (Fig. 6c), VP139 (Fig. 6d), VP137 (Fig. 6e), and VP14 (Fig. 6f) partially colocalized with the MCP in the cytoplasm, suggesting that these mCPs may associate with the MCP to form building blocks in the cytoplasm before the formation of the VF. As observed in the MCP-VP22-cotransfected cells (Fig. 4g), VP22 also mainly distributed in the nucleus at the early stage of SGIV infection (Fig. 6g). Notably, a small aggregation of VP22 in the cytoplasm colocalized with the MCP (Fig. 6g), which implies that VP22 might interact with the MCP after being transported from the nucleus to the cytoplasm.

Furthermore, we also chose several mCP pair combinations for immunofluorescence assays to explore their localization states before the formation of the VF, including VP59 (P4) and VP14 (penton protein), VP59 (P4) and VP137 (P3), and VP59 (P4) and VP38 (P1), with each pair combination being found close to each other in the capsid architecture (Fig. 3d). Interestingly, colocalization between VP59 and VP14 (Fig. 6h), VP59 and VP137 (Fig. 6i), and VP59 and VP38 (Fig. 6j) was not observed, implying the small size of these building blocks in the cytoplasm before the formation of the VF.

## Discussion

A previous cryo-EM structure of the SGIV capsid at 8.6 Å resolution has been reported, in which the MCP, penton protein, anchor protein, and zip protein were observed^32^. The zip protein was thought to be composed of a level component and an arch component^32^. However, due to the resolution limitation and the lack of AA sequence similarities between the mCPs of SGIV and that of other NCVs, such as PBCV-1, their protein sequences or coding genes remained to be identified. Solving the high-resolution structure of the SGIV capsid would provide information on these capsid proteins, identify more targets for vaccine and drug design, and provide clues for exploring the capsid assembly of NCVs. In this study, we determined the cryo-EM structure of the SGIV capsid at 3.5 Å resolution using a block-based reconstruction method^44^. The MCP and seven types of mCPs (penton protein and P1-P6) were identified, and their protein sequences or coding genes were determined. Their atomic models in one ASU were further built, except for P6, which has a short visible polypeptide length in the cryo-EM map. According to CXMS analysis and immunofluorescence assays, VP22 is a strong candidate for P6. However, more experimental data are needed to validate this further.

Previously, only atomic models of PBCV-1 mCPs were reported^37^. In PBCV-1, 14 types of mCPs (including the penton protein) were identified. Penton, TmP, and zip protein were observed in both PBCV-1 and SGIV. However, other types of mCPs differ greatly between these two viruses. For instance, the structural homologs of P4 (VP59) and P2 (VP139) of SGIV were not observed in PBCV-1. Further, two proteins (P9 and P14) of PBCV-1 occupy the position of P4 of SGIV, as well as P8 of PBCV-1 occupying the position of P2 of SGIV. The N-terminal region of TmP between capsomers d and f in SGIV does not exist in the TmP of PBCV-1, and another mCP (P7) occupies the position in PBCV-1. Structural homologs of the anchor protein (P5) and P6 that fill the inner cavity of each pseudo-hexameric capsomer are also not found in PBCV-1. However, five additional types of mCPs (P3, P4, P5, P12, and P13) in the trisymmetron of PBCV-1 were not observed in SGIV. These observations suggest that different viruses may use one or multiple proteins without structural homology to perform similar functions in addition to structural homologs.

Notably, SGIV has an icosahedral inner shell (Fig. 1b), which was observed in the ASFV^29,30,47^ and faustovirus^42^ but not in the PBCV-1^37,99^ structures. ASFV has a bit larger size (~240–250 nm) than SGIV (~228 nm) and PBCV-1 (~190 nm). However, four mCPs were characterized for the ASFV capsid^29,30^. In addition, the genomes of ASFV (~170–194 kbp encoding 150–170 ORFs)^100^ and SGIV (~140-kbp encoding 162 predicted ORFs)^63^ are both much smaller than that of PBCV-1 (~330 kbp genome encoding 416 predicted proteins)^101^. Sixty-three^30^ and sixty-eight^102^ viral protein compositions of ASFV were detected by proteomic analyses. Here, we identified 89 different viral proteins by proteomics for the purified SGIV sample. However, 149 different proteins were detected for PBCV-1^101^. These results suggest that the types of SGIV mCPs could be different from that of PBCV-1 or ASFV.

To date, three capsid assembly models (symmetron model^34^, spiral model^41^, and mCP scaffold model^30^) have been proposed, which are reviewed by Xian and Xiao^99^. Observation of individual symmetrons in the samples of Sericesthis Iridescent Virus^103^ and Tipula Iridescent Virus^104^ support the hypothesis of the proposed symmetron model, in which capsomers are pre-assembled into the trisymmetrons and pentasymmetrons that are then assembled into the icosahedron with the help of the mCPs, such as the zip proteins^34^. However, this model is inconsistent with the continuous assembly process observed in many in situ studies for NCV-infected cells^84,94,105–107^.

The finding that one capsomer in the third layer of the SGIV pentasymmetron displays a 60° rotation compared to the other five capsomers supports the spiral assembly model, where the assembly of the icosahedral capsid initiates from the fivefold vertex, and the capsomers are continuously added in a spiral fashion^41^. This assembly model first illustrated that the fivefold vertex act as the initiation site. Some EM studies for vaccinia virus^105,106^, PBCV-1^84^, and mimivirus^94,107^ display some architectures containing the fivefold vertex in the early VF, supporting this notion. Furthermore, these studies also demonstrate that initiating capsid assembly requires capsid precursors to link with precursors of the inner membrane. Notably, the penton of SGIV was not membrane-associated, and no membrane protein was observed underneath the penton of SGIV. Therefore, mCPs targeting the viral inner membrane surrounding the fivefold vertex may play essential roles in initiating capsid assembly and act as a part of the initiation complex. The penton could be pivotal for gathering these membrane-associated building blocks to form a complete initiation site for capsid assembly. The capsid precursors observed in these EM studies are patches covering the fivefold vertex and surrounding areas, consistent with the above hypothesis.

The cryo-EM structure of ASFV at near-atomic resolution led to the mCP scaffold model^30^. In this model, a number of copies of p17 (one of the mCPs) are assumed to randomly distribute on the inner membrane^30^. After capsid assembly initiates by the p49-penton complex at the fivefold vertex, capsomers are recruited to interact with the p49-penton complex^30^. With the help of TmPs and p17, more capsomers are docked on the membrane surface to form pentasymmetrons and zipper bands, followed by capsomers filling the trisymmetrons to achieve capsid assembly^30^. The functions of mCPs are emphasized in this model. p49 is reported to be located near the capsid vertices^102^ and required for forming the capsid vertices^108^. In the absence of p49, a tubular structure is formed instead of the icosahedral symmetry, indicating that p49 is essential for constructing or stabilizing the icosahedral virus particle^108^.

Although p49 behaves as an integral membrane protein^108^, it may not be the only candidate for initiating the capsid assembly. For instance, p54 of ASFV also plays essential roles at a very early stage of capsid assembly^93^. Our findings demonstrate that VP88 (P5), which is located close to the edge of the pentasymmetron (Fig. 3d, f) and anchored to the viral inner membrane (Fig. 3g), is associated with the ER (Fig. 5a, d). Combined with previous observations^78,84,89–95^, these results indicate that VP88 could function at a very early stage and be pivotal for initiating capsid assembly. Additionally, immunofluorescence assay shows that the SGIV mCPs are complexed with MCP to form various building blocks in the cytoplasm during the early stage of viral infection.

Combining the previous experimental findings mentioned above with our current results allows us to propose a hypothesis for SGIV capsid assembly, in which the MCP and mCPs could be both indispensable and functionally complementary. After SGIV infection, the mCPs and MCP may be expressed and form various building blocks, with free MCP capsomers also existing in the cytoplasm. The VP88-MCP complex could be anchored to the ER membrane by the transmembrane regions of VP88. As the viral assembly continues, the ER membrane collapses, and its cisternal domains, together with the VP88-MCP complex, might be transported into the VF to form the precursor of the inner membrane. The VP14-MCP complex and other mCP-MCP complexes may also be transported into the VF. The VP14-MCP complex possibly gathers these membrane-associated building blocks to form a complete initiation site for capsid assembly. VP22 might be associated with the processes of viral genome condensation and the formation of an inner shell. One end of VP22 may be free outside of the inner shell and pass through precursors of the inner membrane to capture various MCP-mCP complexes and free MCP capsomers. During the formation of pentasymmetrons, the N-terminus of VP137 (TmP) is probably meanwhile fixed. With the help of the VP38-MCP complex, the free ends of the TmP and that of the neighboring pentasymmetrons might be searched, shaped, and fixed step by step, which results in the formation of zipper bands to connect pentasymmetrons. While the zipper band is being formed, free MCP capsomers might be continuously recruited to fill trisymmetrons from the edge of the pentasymmetrons and zipper bands.

The process of NCV capsid assembly is complex and sophisticated. As used in the study of ASFV morphogenesis^93,108^, constructing lethal conditional recombinant viruses would be a strong tool to determine the impact of repression of each capsid protein synthesis on viral morphogenesis and explore their functions in the capsid assembly. Furthermore, future efforts in determining the high-resolution in situ structure of the SGIV virions in the infected cells could provide more insights into capsid assembly.

## Methods

### Cells and virus

EAGS cells were established and maintained in our laboratory^109^, which were grown in Leibovitz’s L15 medium containing 10% fetal bovine serum (Gibco) at 25 °C. SGIV (strain A3/12/98 PPD)^11^ used in this study was kept in our laboratory and propagated in the EAGS cells.

### Cell culture, SGIV propagation, and purification

EAGS cells were plated into flasks (75 cm^2^) the day before infection at ~80% confluence and infected with SGIV at a multiplicity of infection (MOI) of 10. At 48 h post infection, the mixture of supernatant and cells was collected and lysed by three cycles of freezing and thawing. After lysis, the solution was centrifuged at 5000 × *g* for 30 min to remove debris. The supernatant was further ultra-centrifuged at 80,700 × *g* for 2 h using an SW32 Ti rotor (Beckman) to precipitate the virions. The virus-containing pellet was resuspended in PBS overnight and then loaded onto a discontinuous 30–60% (w/v) sucrose (Sigma) density gradient, followed by centrifugation at 139,400 × *g* for 2 h with an SW41 rotor. The bands in the interfaces between 40% and 50% and between 50% and 60% sucrose were extracted using a syringe and diluted with PBS before NP40 detergent was added to the sample to a final concentration of 2%. After incubation overnight at 4 °C, the sample was loaded onto a discontinuous 20–40% (v/v) iodixanol (Sigma) density gradient and centrifuged at 80,000 × *g* for 1 h with an SW41 rotor. The bands in the interfaces between 30% and 35% and between 35% and 40% iodixanol were extracted, resuspended in PBS, and centrifuged at 80,000 × *g* for 1 h to precipitate virions. The pellet was resuspended in 50 μL PBS and used for cryo-EM sample preparation. All steps for purification were performed on ice.

### Cryo-EM sample preparation and data collection

Aliquots (3.0 μL) of purified SGIV were applied to freshly glow-discharged holey carbon-coated copper Quantifoil grids (R2/1 in 300 mesh). The grids were blotted for 3 s and then flash-plunged in liquid ethane using a Vitrobot Mark IV (Thermo Fisher Scientific). The frozen grids were loaded on an FEI Titan Krios microscope (Thermo Fisher Scientific) operated at 300 kV equipped with a Falcon3 camera. Cryo-EM datasets were collected with EPU 2.7 software (Thermo Fisher Scientific) using a magnification of 59,000, yielding a pixel size of 1.4. The defocus range was set from 1.3 to 1.8 μm. Each micrograph was recorded as an image stack composed of 39 frames with an accumulative dose of 50 e^-^/Å^2^ at the exposure time of 1 s. Parameters for data collection are summarized in Supplementary Table 2.

### Cryo-EM data processing

After eliminating micrographs with no particles, a total of 53,306 micrographs were aligned using MotionCor2 v.1.4.2^110^ to correct for beam-induced drift. The contrast transfer function (CTF) parameters were estimated by CTFFIND-4.1^111^. A total of 50,393 micrographs with round shape of the Thon rings and no presence of ice rings in Fourier transformed images were used for the subsequent processing. A total of 71,045 particles were boxed using the EMAN2.3 package^112^, and 6×binned particles (324 pixels, 8.4 Å/pixel) were extracted using RELION-3.0^113^. After reference-free 2D classification, a subset of 70,078 particles was picked to generate an initial model, followed by 3D classification with icosahedral symmetry (I3 symmetry). A total of 37,161 particles from two classes containing an inner shell (Fig. 1b) at 16.8 Å resolution were used to extract the 3×binned particles (648 pixels, 4.2 Å/pixel), and 3D refinement was further performed using a 2,600 Å mask diameter, resulting in a 9.0 Å cryo-EM map. Another class at 16.8 Å resolution without an inner shell was not used for extraction and 3D refinement. To overcome the defocus gradient on giant virions, an ASU of the SGIV virion was clipped into five blocks, and block-based reconstruction^44^ was performed. Unbinned particle images for Block 1, 2, 3 or 5 were extracted using 384 pixels, and those of Block 4 were extracted using 288 pixels. 3D refinement for each block’s unbinned images was performed to generate five block maps with resolutions ranging from 3.7 Å to 3.9 Å. Afterwards, CTF refinement and 3D auto-refine were further performed to improve the quality of the five blocks’ density maps. Finally, each map was sharpened using a mask, which excluded the inner membrane densities, and the final resolutions of the five blocks ranged from 3.4 Å to 3.5 Å. The cryo-EM data processing is summarized in Supplementary Fig. 13, and the FSC curves of five blocks are shown in Supplementary Fig. 14a. Local resolution for the cryo-EM maps of five blocks was estimated using Resmap^114^ (Supplementary Fig. 14b).

### Model building and refinement

The structure of PBCV-1 Vp54 (PDB: 5TIP)^48^ was fitted into the density map of the SGIV MCP with Chimera v.1.14^115^. The model was then manually adjusted in COOT v.0.9.8^116^ to replace its AA sequence using that of VP72 of SGIV. For the penton protein and P1-P5, their Cα models were first manually built with alanine and then mutated to proper amino acids according to sidechain densities using COOT v.0.9.8^116^. The preliminary protein sequences were used to search for the best AA sequence fits from 162 predicted viral proteins encoded by the SGIV genome using the SeqFinder script^37^. After being replaced by the right protein sequences, all atomic models were iteratively refined using COOT v.0.9.8^116^ and PHENIX v.1.19.2^117^. Density maps of five blocks were composited into one map using the vop command in Chimera v.1.14^115^, which would cause some loss in resolution, as described in Chimera’s tutorials. The atomic model for one ASU composed of these capsid proteins with different copies was further built and refined using COOT v.0.9.8^116^ and PHENIX v.1.19.2^117^. The stereochemical quality of the model was evaluated using MolProbity^118^. Statistics for model building and refinement are summarized in Supplementary Table 2. Cryo-EM densities around each capsid protein (MCP, penton protein, and P1-P5) are shown (Supplementary Fig. 14c–i). The original Cryo-EM maps from Block 1 to Block 5 (not the composite density map generated by Chimera v.1.14^115^) were used for density presentions in the figures. Structural figures were prepared using PyMOL v.2.4 (http://www.pymol.org), Chimera v.1.14^115^, and ChimeraX v.1.3^119^.

### Proteomics of the purified SGIV sample

The purified SGIV sample was mixed with loading buffer containing dithiothreitol (DTT, Sigma) and boiled for 10 min at 100 °C. The sample was then loaded onto a 12 % SDS-PAGE gel. When the protein band migrated near the bottom of the stacking gel, i.e., the protein band did not move into the separating gel, it was cut from the gel. Viral protein identification and analysis were then implemented by Novogene Genetics, Beijing, China, and the detailed steps were as follows: The cut gel was destained using destaining buffer (50 mM triethylammonium bicarbonate (TEAB) and 50% acetonitrile (ACN, Thermo Fisher Scientific)) and then dehydrated upon washing with 100% ACN until the gel turned white. Subsequently, it was treated with 1 mL 10 mM DTT for 40 min at 56 °C and alkylated with 1 mL 50 mM iodoacetamide (IAM, Sigma) for 30 min in the dark. The gel was further washed with destaining buffer and treated with ACN as above. 15 μL 10 ng/µL trypsin (Promega) was added into the gel and incubation for 30 min on ice, then made up to 100 μL with 100 mM TEAB. Proteins were digested overnight at 37 °C. After centrifugation at a low speed, the supernatant was collected. Subsequently, 200 µL of ACN was added to the gel, followed by vortex mixing, and the remaining peptides were extracted in 100 μL of 0.1% formic acid (FA, Thermo Fisher Scientific). Extracted supernatants were combined, centrifuged at 12,000 × *g* for 5 min at room temperature and then lyophilized. The powder was dissolved and mixed in 0.1% FA. The supernatant was slowly loaded onto a C18 desalting column (Thermo Fisher Scientific), washed with 1 mL of washing solution (0.1% FA and 4% ACN) three times, and then eluted twice with 0.4 mL of elution buffer (0.1% FA and 75% ACN). The eluents were collected, combined, and lyophilized. Mobile phase A (0.1% FA) and B (80% ACN and 0.1% FA) solutions were prepared. The lyophilized powder was dissolved in 10 μL of solution A, centrifuged at 14,000 × *g* for 20 min at 4 °C, and 1 μg of the sample was injected into a home-made C18 Nano-Trap column (4.5 cm × 75 μm, 3 μm). Peptides were separated in a home-made analytical column (15 cm × 150 μm, 1.9 μm) using linear gradient elution on an EASY-nLC^TM^ 1200 UHPLC system (Thermo Fisher Scientific). The separated peptides were analyzed using a Q Exactive^TM^ HF-X mass spectrometer (Thermo Fisher Scientific) with ion source of Nanospray Flex™ (ESI), spray voltage of 2.3 kV, and ion transport capillary temperature of 320 °C. The full scan ranged from *m*/*z* 350 to 1500 with resolution of 60,000 (at *m*/*z* 200), the automatic gain control (AGC) target value was 3 × 10^6^, and the maximum ion injection time was 20 ms. The top 40 precursors of the most abundant species in the full scan were selected and fragmented by higher energy collisional dissociation (HCD) and analyzed in MS/MS, where the resolution was 15,000 (at *m*/*z* 200), the AGC target value was 1 × 10^5^, the maximum ion injection time was 45 ms, the normalized collision energy was set as 27%, the intensity threshold was 2.2 × 10^4^, and the dynamic exclusion parameter was 20 s. The MS/MS raw data were directly imported into the Proteome Discoverer 2.2 software (Thermo Fisher Scientific) for database searching, peptide spectrum matches (PSMs), and protein quantification. A fasta file bearing 162 annotated SGIV protein sequences^63^ was used as the database. The search parameters were set as previously described^120^. Enzyme specificity was set to trypsin. Searches were performed using a 10-ppm precursor mass tolerance and 0.02-Da fragment mass tolerance. The search included N-termini (+42.011 Da), and carbamidomethylation of cysteine residues (+57.021 Da) was set as a static modification, while oxidation of methionine residues (+15.995 Da) was set as a dynamic modification. Up to two missed cleavage sites were allowed for protease digestion, and peptides had to be fully tryptic. To improve the quality of the results and reduce the false positive rate, Proteome Discoverer 2.2 software (Thermo Fisher Scientific) was further used to filter the results. PSMs with a credibility of > 99% and proteins containing at least one unique peptide segment were retained and performed with ≤1.0% FDR. Gene Ontology (GO) and InterPro (IPR) functional analyses were conducted using the InterProScan 5 software against non-redundant protein databases (including Pfam, PRINTS, ProDom, SMART, ProSiteProfiles, PANTHER)^121^, and the databases of Clusters of Orthologous Groups (COG) and Kyoto Encyclopedia of Genes and Genomes (KEGG) were used to analyze the protein family and pathway.

### CXMS analysis

The purified SGIV sample was freeze-thawed 10 times and then crosslinked with bis[sulfosuccinimidyl] suberate (BS^3^, Thermo Fisher Scientific) at a final cross-linker concentration of 5 mM; 20 mM Tris was used to terminate the reaction after incubation at room temperature for 1 h. The cross-linked sample was precipitated with pre-chilled acetone and lyophilized for mass spectrometry (MS) analysis. The pellet was dissolved in 8 M urea and 100 mM Tris (pH 8.5), followed by TCEP reduction, IAM alkylation, and trypsin (Pierce) digestion. Digestion was quenched with 5% FA, the peptides were desalted with a MonoSpin C18 (GL Sciences), and then separated within a home-packed C18 column (75 μm × 25 cm, 1.9 μm) on an EASY-nLC^TM^ 1200 UHPLC system (Thermo Fisher Scientific) using a 0–80% ACN gradient elution. Mobile phases A and B were water and ACN with 0.1% FA. The %B was linearly increased from 5–28% over 100 min, followed by an increase to 45% within 10 min and a further increase to 100% within 5 min before the last 5 min 100% B process. The samples were analyzed on a liquid chromatography system coupled to a Q-Exactive HF-X mass spectrometer (Thermo Fisher Scientific). The method parameters of the run were as follows: data-dependent acquisition; full MS resolution 60,000; MS1 AGC target 3e6; MS1 maximum IT 50 ms; scan range from 300–1800; dd-MS/MS resolution 15,000; MS/MS AGC target 5e5; MS2 maximum IT 40 ms; loop count top 30; isolation window 1.7; MS2 minimum AGC target 8e3; charge exclusion: unassigned, 1, 8, >8; peptide match off; exclude isotope on; and dynamic exclusion 30 s. Raw data were processed with pLink 2 software^122^. The trypsin KR-C was chosen as the enzyme with up to three missed cleavages specified; cross-linker was set as BS^3^. The precursor tolerance was limited to 20 ppm, and the fragment tolerance was set to 25 ppm. MS/MS spectra were matched against the Singapore grouper iridovirus database (A fasta file bearing 162 annotated SGIV protein sequences^63^). Carbamidomethyl-cysteine residues were defined as a fixed modification, and methionine oxidation was defined as a variable modification. A 5% FDR was applied at the peptide-spectrum match (PSM) level. All of the crosslinking results were manually checked.

### Fluorescence colocalization analysis of mCPs with the MCP in cotransfected cells

DNA encoding full-length VP88, VP59, VP38, VP139, VP137, VP14, or VP22 was separately cloned into the pEGFP-C1 or pEGFP-N3 vector, resulting in recombinant plasmids (pEGFP-VP88, pEGFP-VP59, pEGFP-VP38, pEGFP-VP139, pEGFP-VP137, pEGFP-VP14, and pEGFP-VP22) expressing the mCP fused to enhanced green fluorescent protein (EGFP). The DNA encoding full-length MCP was cloned into the pDsRed-N1 plasmid, resulting in a recombinant plasmid (pDsRed-MCP) expressing the MCP fused to DsRed. The colocalization relationships between mCPs and the MCP were determined by cotransfection with pDsRed-MCP and a recombinant mCP plasmid (pEGFP-VP88, pEGFP-VP59, pEGFP-VP38, pEGFP-VP139, pEGFP-VP137, pEGFP-VP14, or pEGFP-VP22) into EAGS cells. Briefly, EAGS cells were seeded into glass-bottom cell culture dishes (35 mm) for 18 h and then cotransfected with 0.5 μg of pDsRed-MCP plasmid and 0.5 μg of the recombinant mCP plasmid using Lipofectamine 2000 (Invitrogen) according to the manufacturer’s instructions. At 48 h post transfection (h p.t.), cells were fixed, stained with DAPI (Sigma), and then observed by confocal laser scanning microscopy (Carl Zeiss). The confocal images were analyzed using the Zeiss ZEN 2012 image analysis software.

### Fluorescence colocalization analysis of three mCPs with the ER

The subcellular localization of the predicted membrane proteins (VP88, VP38, and VP59) was determined by cotransfection with the pDsRed2-ER (an ER-specific marker, Clontech) plasmid and the recombinant mCP plasmid (pEGFP-VP88, pEGFP-VP59, or pEGFP-VP38) into EAGS cells. Briefly, EAGS cells were seeded into glass-bottom cell culture dishes (35 mm) for 18 h and then cotransfected with 0.5 μg of the corresponding recombinant plasmid and 0.5 μg of pDsRed2-ER plasmid using Lipofectamine 2000 (Invitrogen). At 24 h p.t., cells were infected with/without SGIV for another 24 h. After fixation, cells were stained with DAPI and then observed by confocal laser scanning microscopy. The confocal images were analyzed using the Zeiss ZEN 2012 image analysis software.

### Immunofluorescence assay

The intracellular localization of the identified capsid proteins during SGIV infection was determined using an immunofluorescence assay^109^. In brief, EAGS cells were seeded into glass-bottom cell culture dishes (35 mm) for 18 h and then incubated with SGIV for 12 h and 24 h. After fixation and permeabilization, cells were incubated with the mouse monoclonal antibody (mAb) against SGIV MCP (diluted at a ratio of 1:1200) and rabbit polyclonal antibody against SGIV VP88 (VP59, VP38, VP139, VP137, VP14, or VP22, diluted at a ratio of 1:800) for 1 h. Anti-rabbit Alexa Fluor 555 secondary antibody (diluted at a ratio of 1:600, Thermo Fisher Scientific, A-21428) and anti-mouse Alexa Fluor 488 secondary antibody (diluted at a ratio of 1:600, Thermo Fisher Scientific, A-11001) were used. For the cells incubated with SGIV for 12 h, they were also incubated with a mouse polyclonal antibody against VP59 (diluted at a ratio of 1:800) and a rabbit polyclonal antibody against SGIV VP14 (VP137 or VP38, diluted at a ratio of 1:800), and the corresponding secondary antibodies described above were then used. All the primary antibodies used here were produced by Wuhan Genecreate Biological Engineering Co. Ltd (Wuhan, China). Finally, cells were stained with DAPI and observed by confocal laser scanning microscopy. The confocal images were analyzed using the Zeiss ZEN 2012 image analysis software.

### Reporting summary

Further information on research design is available in the Nature Portfolio Reporting Summary linked to this article.

## Supplementary information

Supplementary Information

Reporting Summary

## Data Availability

The atomic structure coordinates of one ASU from the SGIV capsid have been deposited in the RCSB Protein Data Bank (PDB) under the accession code 8HIF. The cryo-EM maps of the SGIV intact virion (a 3×binned map), five blocks (block 1, block 2, block 3, block 4, and block 5), and a composite density map generated by combining the density maps of five blocks have been deposited in the Electron Microscopy Data Bank (EMDB) with the accession codes EMD-34251, EMD-34227, EMD-34230, EMD-34235, EMD-34229, EMD-34236, and EMD-34815. Other density maps and atomic structures for analysis, including EMD-1580, EMD-0436, EMD-8144, EMD-0815, EMD-8748, 6G45, 5TIP, 3J26, 5OAC, 2VVF, 1CJD, 2BBD, 2YGB, 6L2T, 5J7O, 6B1T, 6NCL, 1W8X, 6G42, 3J26, and 1X9P, were obtained from the EMDB and PDB. The MS data for the proteomics and CXMS analyses have been deposited into the ProteomeXchange Consortium via the PRIDE partner repository with the dataset identifier PXD038093.

## References

[1] Koonin EV, Yutin N (2019). Evolution of the large nucleocytoplasmic DNA viruses of eukaryotes and convergent origins of viral gigantism. Adv. Virus Res..

[2] Koonin EV, Yutin N (2018). Multiple evolutionary origins of giant viruses. F1000Res..

[3] Krupovic M, Koonin EV (2015). Polintons: a hotbed of eukaryotic virus, transposon and plasmid evolution. Nat. Rev. Microbiol..

[4] Koonin, E. V. et al. Global organization and proposed megataxonomy of the virus world. *Microbiol. Mol. Biol. Rev*. **84**, e00061–19 (2020). 10.1128/MMBR.00061-19 PMC7062200 32132243

[5] Yutin N, Koonin EV (2012). Hidden evolutionary complexity of nucleo-cytoplasmic large DNA viruses of eukaryotes. Virol. J..

[6] Chinchar VG (2017). ICTV virus taxonomy profile: iridoviridae. J. Gen. Virol..

[7] Chinchar VG, Waltzek TB, Subramaniam K (2017). Ranaviruses and other members of the family Iridoviridae: their place in the virosphere. Virology.

[8] İnce İA, Özcan O, Ilter-Akulke AZ, Scully ED, Özgen A (2018). Invertebrate Iridoviruses: a glance over the last decade. a glance over the last decade. Viruses.

[9] Daszak P (1999). Emerging infectious diseases and amphibian population declines. Emerg. Infect. Dis..

[10] Hyatt AD (2000). Comparative studies of piscine and amphibian iridoviruses. Arch. Virol..

[11] Qin QW (2003). Characterization of a novel ranavirus isolated from grouper Epinephelus tauvina. Dis. Aquat. Org..

[12] Gray MJ, Miller DL, Hoverman JT (2009). Ecology and pathology of amphibian ranaviruses. Dis. Aquat. Org..

[13] Kurita J, Nakajima K (2012). Megalocytiviruses. Viruses.

[14] Walker PJ, Winton JR (2010). Emerging viral diseases of fish and shrimp. Vet. Res..

[15] Whittington RJ, Becker JA, Dennis MM (2010). Iridovirus infections in finfish - critical review with emphasis on ranaviruses. J. Fish. Dis..

[16] Marschang, R. E. *Viruses infecting reptiles. Viruses* **3**, 2087–2126 (2011). 10.3390/v3112087 PMC3230843 22163336

[17] Qin Q (2001). Electron microscopic observations of a marine fish iridovirus isolated from brown-spotted grouper, Epinephelus tauvina. J. Virol. Methods.

[18] Huang X (2011). Singapore grouper iridovirus, a large DNA virus, induces nonapoptotic cell death by a cell type dependent fashion and evokes ERK signaling. Apoptosis.

[19] Kondylis P, Schlicksup CJ, Zlotnick A, Jacobson SC (2019). Analytical techniques to characterize the structure, properties, and assembly of virus capsids. Anal. Chem..

[20] Schlicksup CJ (2018). Hepatitis B virus core protein allosteric modulators can distort and disrupt intact capsids. eLife.

[21] Schwarz B, Uchida M, Douglas T (2017). Biomedical and catalytic opportunities of virus-like particles in nanotechnology. Adv. Virus Res..

[22] Luque D, Castón JR (2020). Cryo-electron microscopy for the study of virus assembly. Nat. Chem. Biol..

[23] Sodeik B, Krijnse-Locker J (2002). Assembly of vaccinia virus revisited: de novo membrane synthesis or acquisition from the host?. Trends Microbiol..

[24] Asgari S (2017). ICTV virus taxonomy profile: ascoviridae. J. Gen. Virol..

[25] Philippe N (2013). Pandoraviruses: amoeba viruses with genomes up to 2.5 Mb reaching that of parasitic eukaryotes. Science.

[26] Legendre M (2014). Thirty-thousand-year-old distant relative of giant icosahedral DNA viruses with a pandoravirus morphology. Proc. Natl Acad. Sci. USA.

[27] Christo-Foroux, E. et al. Characterization of Mollivirus kamchatka, the first modern representative of the proposed molliviridae family of giant viruses. *J. Virol*. **94**, e01997–19 (2020). 10.1128/JVI.01997-19 PMC7108836 31996429

[28] Legendre M (2015). In-depth study of Mollivirus sibericum, a new 30,000-y-old giant virus infecting Acanthamoeba. Proc. Natl Acad. Sci. USA.

[29] Liu S (2019). Cryo-EM structure of the African swine fever virus. Cell Host Microbe.

[30] Wang N (2019). Architecture of African swine fever virus and implications for viral assembly. Science.

[31] Andrés G, Charro D, Matamoros T, Dillard RS, Abrescia NGA (2020). The cryo-EM structure of African swine fever virus unravels a unique architecture comprising two icosahedral protein capsids and two lipoprotein membranes. J. Biol. Chem..

[32] Pintilie G (2019). Segmentation and comparative modeling in an 8.6-Å Cryo-EM map of the Singapore grouper iridovirus. Structure.

[33] Yan X (2000). Structure and assembly of large lipid-containing dsDNA viruses. Nat. Struct. Biol..

[34] Yan X (2009). The capsid proteins of a large, icosahedral dsDNA virus. J. Mol. Biol..

[35] Cherrier MV (2009). An icosahedral algal virus has a complex unique vertex decorated by a spike. Proc. Natl Acad. Sci. USA.

[36] Zhang X (2011). Three-dimensional structure and function of the Paramecium bursaria chlorella virus capsid. Proc. Natl Acad. Sci. USA.

[37] Fang, Q. et al. Near-atomic structure of a giant virus. *Nat. Commun*. **10**, 388 (2019). 10.1038/s41467-019-08319-6 PMC6344570 30674888

[38] Xiao, C. et al. Structural studies of the giant mimivirus. *PLoS Biol*. **7**, e1000092 (2009). 10.1371/journal.pbio.1000092 PMC2671561 19402750

[39] Yan X, Chipman PR, Castberg T, Bratbak G, Baker TS (2005). The marine algal virus PpV01 has an icosahedral capsid with T=219 quasisymmetry. J. Virol..

[40] Okamoto K (2018). Cryo-EM structure of a Marseilleviridae virus particle reveals a large internal microassembly. Virology.

[41] Xiao C (2017). Cryo-EM reconstruction of the Cafeteria roenbergensis virus capsid suggests novel assembly pathway for giant viruses. Sci. Rep..

[42] Klose T (2016). Structure of faustovirus, a large dsDNA virus. Proc. Natl Acad. Sci. USA.

[43] Andreani J (2017). Pacmanvirus, a new giant icosahedral virus at the crossroads between Asfarviridae and Faustoviruses. J. Virol..

[44] Zhu D (2018). Pushing the resolution limit by correcting the Ewald sphere effect in single-particle Cryo-EM reconstructions. Nat. Commun..

[45] Leong PA, Yu X, Zhou ZH, Jensen GJ (2010). Correcting for the ewald sphere in high-resolution single-particle reconstructions. Methods Enzymol..

[46] Zhang X, Zhou ZH (2011). Limiting factors in atomic resolution cryo electron microscopy: no simple tricks. J. Struct. Biol..

[47] Salas ML, Andrés G (2013). African swine fever virus morphogenesis. Virus Res..

[48] Castro Cde (2018). Structure of the chlorovirus PBCV-1 major capsid glycoprotein determined by combining crystallographic and carbohydrate molecular modeling approaches. Proc. Natl Acad. Sci. USA.

[49] Bahar MW, Graham SC, Stuart DI, Grimes JM (2011). Insights into the evolution of a complex virus from the crystal structure of vaccinia virus D13. Structure.

[50] Liu Q (2019). Structure of the African swine fever virus major capsid protein p72. Cell Res..

[51] Laanto E (2017). Virus found in a boreal lake links ssDNA and dsDNA viruses. Proc. Natl Acad. Sci. USA.

[52] Abrescia NGA (2008). Insights into virus evolution and membrane biogenesis from the structure of the marine lipid-containing bacteriophage PM2. Mol. Cell.

[53] Benson SD, Bamford JK, Bamford DH, Burnett RM (1999). Viral evolution revealed by bacteriophage PRD1 and human adenovirus coat protein structures. Cell.

[54] Born D (2018). Capsid protein structure, self-assembly, and processing reveal morphogenesis of the marine virophage mavirus. Proc. Natl Acad. Sci. USA.

[55] Zhang X (2012). Structure of Sputnik, a virophage, at 3.5-Å resolution. Proc. Natl Acad. Sci. USA.

[56] Dai X, Wu L, Sun R, Zhou ZH (2017). Atomic structures of minor proteins VI and VII in human adenovirus. J. Virol..

[57] Roberts MM, White JL, Grütter MG, Burnett RM (1986). Three-dimensional structure of the adenovirus major coat protein hexon. Science.

[58] Khayat R (2005). Structure of an archaeal virus capsid protein reveals a common ancestry to eukaryotic and bacterial viruses. Proc. Natl Acad. Sci. USA.

[59] Xiao C, Rossmann MG (2011). Structures of giant icosahedral eukaryotic dsDNA viruses. Curr. Opin. Virol..

[60] Burnett RM, Grütter MG, White JL (1985). The structure of the adenovirus capsid. J. Mol. Biol..

[61] Klose T, Rossmann MG (2014). Structure of large dsDNA viruses. Biol. Chem..

[62] Xian Y, Karki CB, Silva SM, Li L, Xiao C (2019). The roles of electrostatic interactions in capsid assembly mechanisms of giant viruses. Int. J. Mol. Sci..

[63] Yao D (2019). In-depth proteomic profiling of the Singapore grouper iridovirus virion. Arch. Virol..

[64] Abrescia NGA (2004). Insights into assembly from structural analysis of bacteriophage PRD1. Nature.

[65] Zubieta C, Schoehn G, Chroboczek J, Cusack S (2005). The structure of the human adenovirus 2 penton. Mol. Cell.

[66] Cockburn JJB (2004). Membrane structure and interactions with protein and DNA in bacteriophage PRD1. Nature.

[67] Xian Y, Avila R, Pant A, Yang Z, Xiao C (2021). The role of tape measure protein in nucleocytoplasmic large DNA virus capsid assembly. Viral Immunol..

[68] Ravanello MP, Hruby DE (1994). Conditional lethal expression of the vaccinia virus L1R myristylated protein reveals a role in virion assembly. J. Virol..

[69] Rodríguez JR, Risco C, Carrascosa JL, Esteban M, Rodríguez D (1997). Characterization of early stages in vaccinia virus membrane biogenesis: implications of the 21-kilodalton protein and a newly identified 15-kilodalton envelope protein. J. Virol..

[70] Bryant M, Ratner L (1990). Myristoylation-dependent replication and assembly of human immunodeficiency virus 1. Proc. Natl Acad. Sci. USA.

[71] Göttlinger HG, Sodroski JG, Haseltine WA (1989). Role of capsid precursor processing and myristoylation in morphogenesis and infectivity of human immunodeficiency virus type 1. Proc. Natl Acad. Sci. USA.

[72] Gheysen D (1989). Assembly and release of HIV-1 precursor Pr55gag virus-like particles from recombinant baculovirus-infected insect cells. Cell.

[73] Rein A, McClure MR, Rice NR, Luftig RB, Schultz AM (1986). Myristylation site in Pr65gag is essential for virus particle formation by Moloney murine leukemia virus. Proc. Natl Acad. Sci. USA.

[74] Chow M (1987). Myristylation of picornavirus capsid protein VP4 and its structural significance. Nature.

[75] Altschul SF, Gish W, Miller W, Myers EW, Lipman DJ (1990). Basic local alignment search tool. J. Mol. Biol..

[76] Mount DW (2007). Using the basic local alignment search tool (BLAST). CSH Protoc..

[77] Robert X, Gouet P (2014). Deciphering key features in protein structures with the new ENDscript server. Nucleic Acids Res..

[78] Zhao Z (2008). Identification and characterization of a novel envelope protein in Rana grylio virus. J. Gen. Virol..

[79] Sung MT, Cao TM, Coleman RT, Budelier KA (1983). Gene and protein sequences of adenovirus protein VII, a hybrid basic chromosomal protein. Proc. Natl Acad. Sci. USA.

[80] Wiethoff CM, Wodrich H, Gerace L, Nemerow GR (2005). Adenovirus protein VI mediates membrane disruption following capsid disassembly. J. Virol..

[81] Maier O, Galan DL, Wodrich H, Wiethoff CM (2010). An N-terminal domain of adenovirus protein VI fragments membranes by inducing positive membrane curvature. Virology.

[82] Novoa RR (2005). Virus factories: associations of cell organelles for viral replication and morphogenesis. Biol. Cell.

[83] Fischer MG (2011). Sputnik and Mavirus: more than just satellite viruses. Nat. Rev. Microbiol..

[84] Milrot E (2016). Virus-host interactions: insights from the replication cycle of the large Paramecium bursaria chlorella virus. Cell. Microbiol..

[85] Sodeik B (1993). Assembly of vaccinia virus: role of the intermediate compartment between the endoplasmic reticulum and the Golgi stacks. J. Cell Biol..

[86] Suárez C (2013). Open membranes are the precursors for assembly of large DNA viruses. Cell. Microbiol..

[87] Suzan-Monti M, La Scola B, Barrassi L, Espinosa L, Raoult D (2007). Ultrastructural characterization of the giant volcano-like virus factory of Acanthamoeba polyphaga Mimivirus. PLoS ONE.

[88] Paperna I, Ilana Sabnai H, Colorni A (1982). An outbreak of lymphocystis in Sparus aurata L. in the Gulf of Aqaba, Red Sea. J. Fish. Dis..

[89] Liu Y (2016). Visualization of assembly intermediates and budding vacuoles of Singapore grouper iridovirus in grouper embryonic cells. Sci. Rep..

[90] Andrés G, García-Escudero R, Simón-Mateo C, Viñuela E (1998). African swine fever virus is enveloped by a two-membraned collapsed cisterna derived from the endoplasmic reticulum. J. Virol..

[91] Rouiller I, Brookes SM, Hyatt AD, Windsor M, Wileman T (1998). African swine fever virus is wrapped by the endoplasmic reticulum. J. Virol..

[92] Cobbold C, Whittle JT, Wileman T (1996). Involvement of the endoplasmic reticulum in the assembly and envelopment of African swine fever virus. J. Virol..

[93] Rodríguez JM, García-Escudero R, Salas ML, Andrés G (2004). African swine fever virus structural protein p54 is essential for the recruitment of envelope precursors to assembly sites. J. Virol..

[94] Mutsafi Y, Shimoni E, Shimon A, Minsky A (2013). Membrane assembly during the infection cycle of the giant Mimivirus. PLoS Pathog..

[95] Whitley DS (2010). Frog virus 3 ORF 53R, a putative myristoylated membrane protein, is essential for virus replication in vitro. Virology.

[96] Kelley LA, Mezulis S, Yates CM, Wass MN, Sternberg MJE (2015). The Phyre2 web portal for protein modeling, prediction and analysis. Nat. Protoc..

[97] Krogh A, Larsson B, Heijne Gvon, Sonnhammer EL (2001). Predicting transmembrane protein topology with a hidden Markov model: application to complete genomes. J. Mol. Biol..

[98] Sonnhammer EL, Heijne Gvon, Krogh A (1998). A hidden Markov model for predicting transmembrane helices in protein sequences. Proc. Int. Conf. Intell. Syst. Mol. Biol..

[99] Xian Y, Xiao C (2020). Current capsid assembly models of icosahedral nucleocytoviricota viruses. Adv. Virus Res..

[100] Cackett G (2020). The African Swine Fever Virus Transcriptome. J. Virol..

[101] Dunigan DD (2012). Paramecium bursaria chlorella virus 1 proteome reveals novel architectural and regulatory features of a giant virus. J. Virol..

[102] Alejo A, Matamoros T, Guerra M, Andrés G (2018). A proteomic atlas of the African swine fever virus particle. J. Virol..

[103] Wrigley NG (1969). An electron microscope study of the structure of Sericesthis iridescent virus. J. Gen. Virol..

[104] Wrigley NG (1970). An electron microscope study of the structure of Tipula iridescent virus. J. Gen. Virol..

[105] Chlanda P, Carbajal MA, Cyrklaff M, Griffiths G, Krijnse-Locker J (2009). Membrane rupture generates single open membrane sheets during vaccinia virus assembly. Cell Host Microbe.

[106] Krijnse Locker J, Chlanda P, Sachsenheimer T, Brügger B (2013). Poxvirus membrane biogenesis: rupture not disruption. Cell. Microbiol..

[107] Kuznetsov YG, Klose T, Rossmann M, McPherson A (2013). Morphogenesis of mimivirus and its viral factories: an atomic force microscopy study of infected cells. J. Virol..

[108] Epifano C, Krijnse-Locker J, Salas ML, Salas J, Rodríguez JM (2006). Generation of filamentous instead of icosahedral particles by repression of African swine fever virus structural protein pB438L. J. Virol..

[109] Huang X, Huang Y, Sun J, Han X, Qin Q (2009). Characterization of two grouper Epinephelus akaara cell lines: Application to studies of Singapore grouper iridovirus (SGIV) propagation and virus–host interaction. Application to studies of Singapore grouper iridovirus (SGIV) propagation and virus-host interaction. Aquaculture.

[110] Zheng SQ (2017). MotionCor2: anisotropic correction of beam-induced motion for improved cryo-electron microscopy. Nat. Methods.

[111] Rohou A, Grigorieff N (2015). CTFFIND4: Fast and accurate defocus estimation from electron micrographs. Fast and accurate defocus estimation from electron micrographs. J. Struct. Biol..

[112] Tang G (2007). EMAN2: an extensible image processing suite for electron microscopy. J. Struct. Biol..

[113] Zivanov J (2018). New tools for automated high-resolution cryo-EM structure determination in RELION-3. eLife.

[114] Kucukelbir A, Sigworth FJ, Tagare HD (2014). Quantifying the local resolution of cryo-EM density maps. Nat. Methods.

[115] Pettersen EF (2004). UCSF Chimera—a visualization system for exploratory research and analysis. J. Comput. Chem..

[116] Emsley P, Lohkamp B, Scott WG, Cowtan K (2010). Features and development of Coot. Acta Crystallogr. D. Biol. Crystallogr..

[117] Adams PD (2010). PHENIX: a comprehensive Python-based system for macromolecular structure solution. Acta Crystallogr. D. Biol. Crystallogr..

[118] Chen VB (2010). MolProbity: all-atom structure validation for macromolecular crystallography. Acta Crystallogr. D. Biol. Crystallogr..

[119] Goddard TD (2018). UCSF ChimeraX: meeting modern challenges in visualization and analysis. Protein Sci..

[120] Luo Y (2020). Phosphoproteomics and proteomics reveal metabolism as a key node in LPS-induced acute inflammation in RAW264.7. Inflammation.

[121] Jones P (2014). InterProScan 5: genome-scale protein function classification. Bioinformatics.

[122] Chen Z-L (2019). A high-speed search engine pLink 2 with systematic evaluation for proteome-scale identification of cross-linked peptides. Nat. Commun..

