# Supplementary material for: Near-atomic architecture of Singapore grouper iridovirus and implications for giant virus assembly
Source: Nat Commun. 2023 Apr 12;14:2050. doi: 10.1038/s41467-023-37681-9 (PMC10090177; doi:10.1038/s41467-023-37681-9)
Supplement: Supplementary file 1 — Supplementary Information [file 41467_2023_37681_MOESM1_ESM.pdf]

Supplementary Information

Near-atomic architecture of Singapore grouper iridovirus and implications for giant virus assembly

Zhennan Zhao, et al

Supplementary Figures 1-14

Supplementary Tables 1-2

Supplementary References

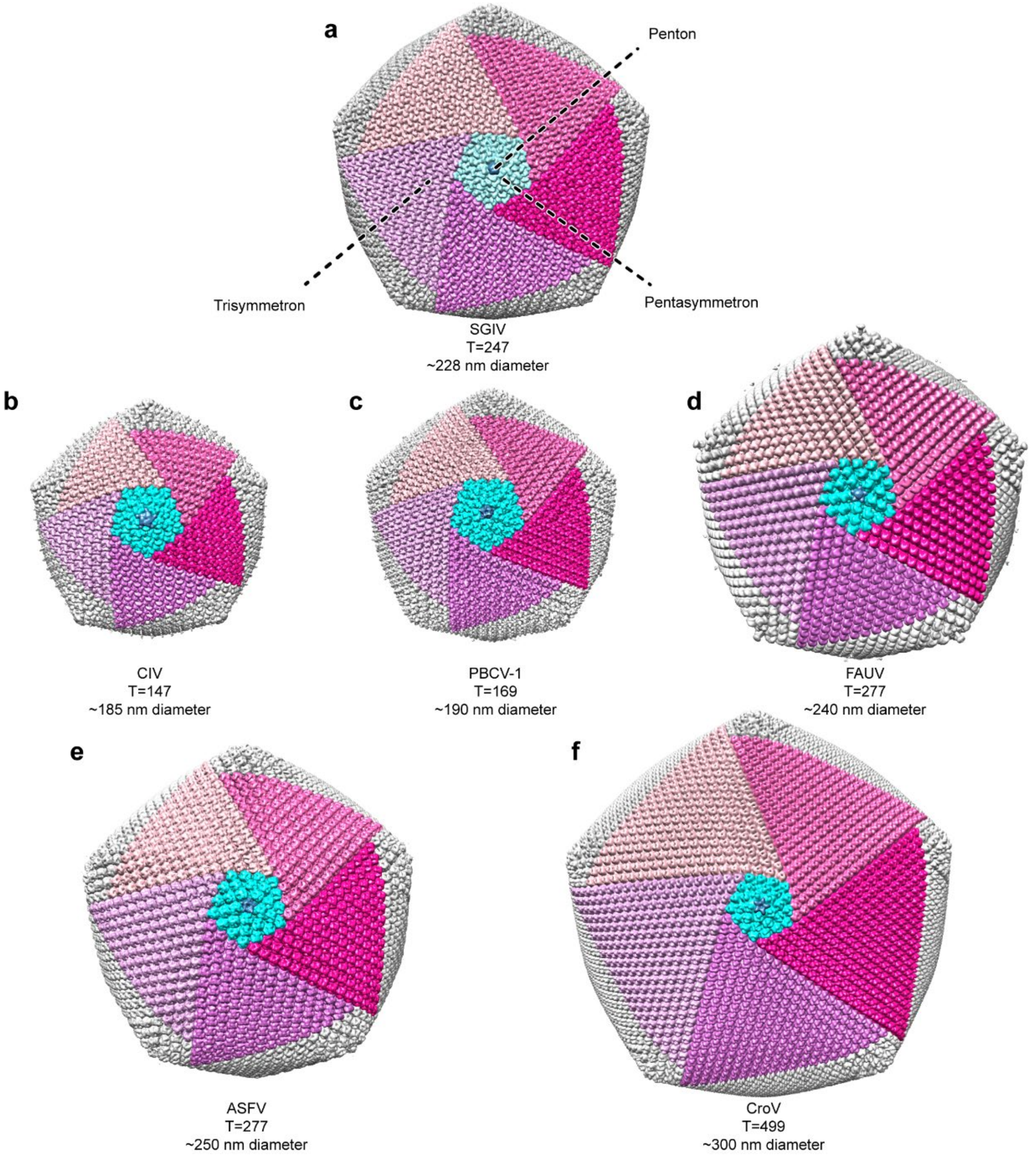

Supplementary Fig. 1 | Overall structure of icosahedral NCV virions. One pentasymmetron is colored in cyan, and five trisymmetrons are colored in deep pink, hot pink, pink, plum, and orchid, respectively. The penton at the five-fold vertex is colored in steel blue. The cryo-EM maps of SGIV (this paper) (**a**), CIV (EMDB: EMD-1580)[1] (**b**), PBCV-1 (EMDB: EMD-0436)[2] (**c**), FAUV (EMDB: EMD-8144)[3] (**d**), ASFV (EMDB: EMD-0815)[4] (**e**), and CroV (EMDB: EMD-8748)[5] (**f**) are used for presentation.

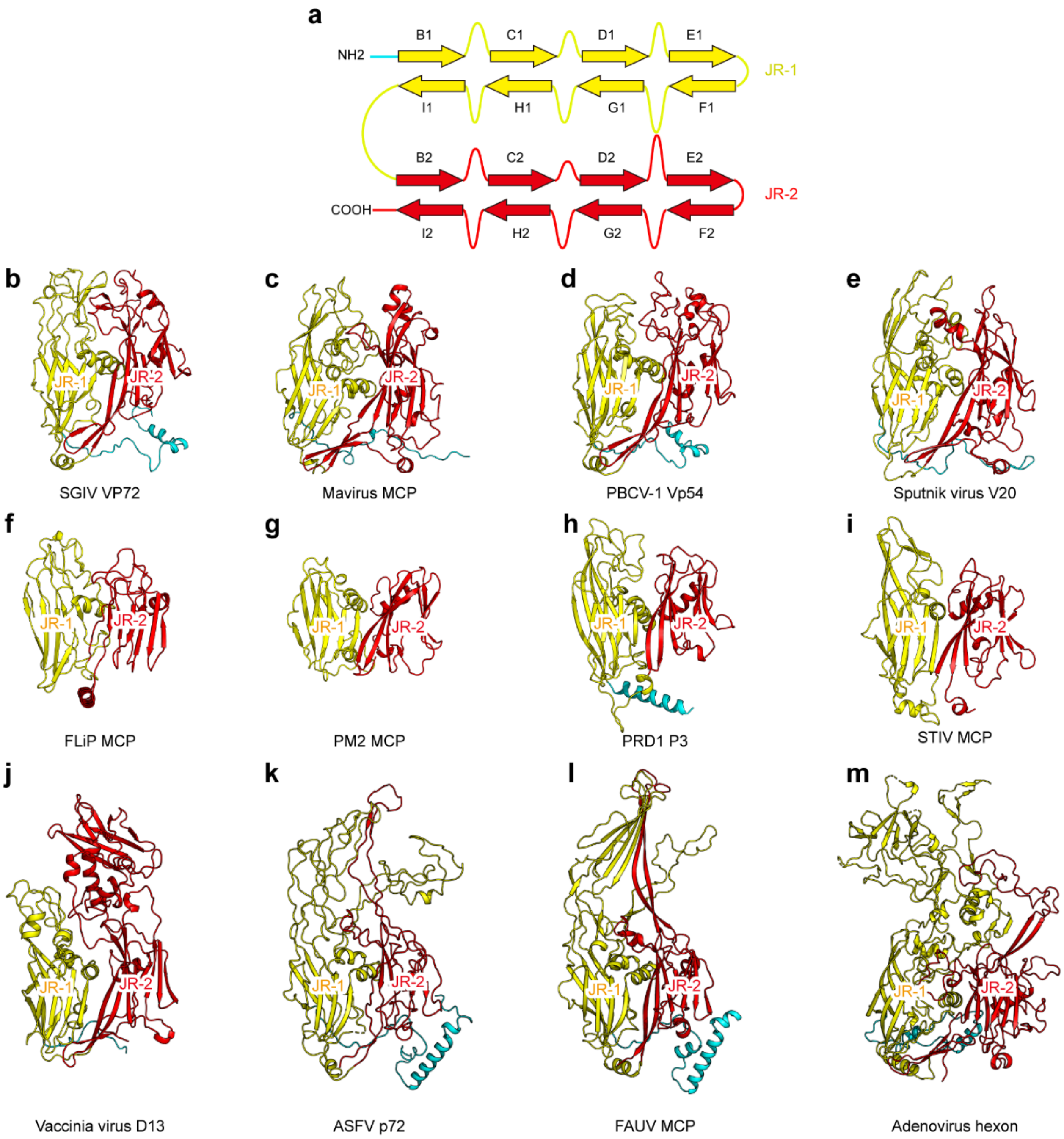

Supplementary Fig. 2 | Structural comparison of MCPs with a double JR fold. a Schematic diagram of the double JR fold. Two JR domains (JR-1 and JR-2) are colored in yellow and red, respectively, with the β-strands shown as arrows and insertions shown as lines. The N-terminal base is colored in cyan, and the C-terminus is colored in orange. **b-m** Structures of MCPs with a double JR fold in SGIV (this paper), Mavirus (PDB: 6G45)[6], PBCV-1 (PDB: 5TIP)[7], Sputnik virus (PDB: 3J26)[8], *Flavobacterium*-infecting, lipid-containing phage (FLiP) (PDB: 5OAC)[9], PM2 (PDB: 2VVF)[10], PRD-1 (PDB: 1CJD)[11], STIV (PDB: 2BBD)[12], vaccinia virus (PDB: 2YGB)[13], ASFV (PDB: 6L2T)[4], FAUV (PDB: 5J7O)[3], and AdV (PDB: 6B1T)[14].

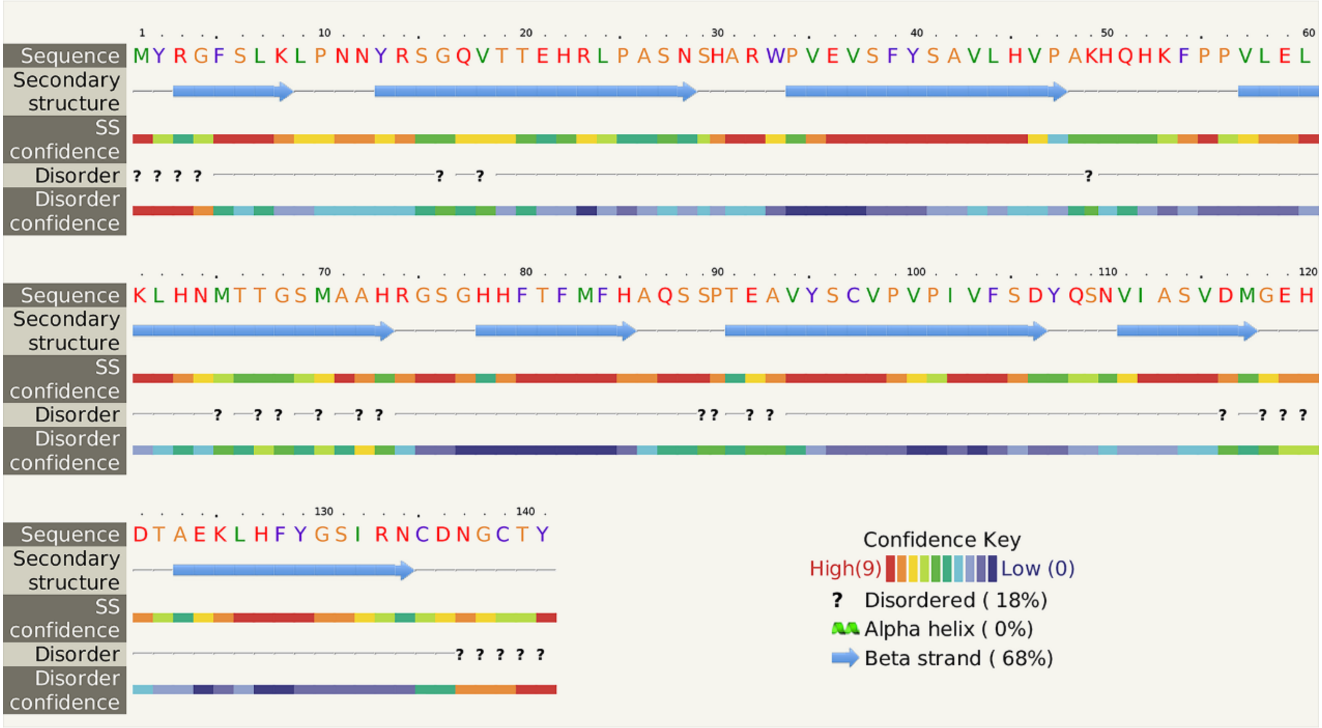

Supplementary Fig. 3 | Secondary structure prediction of VP14. The PHYRE2 Protein Fold Recognition Server[15] was used to predict the secondary structure of VP14.

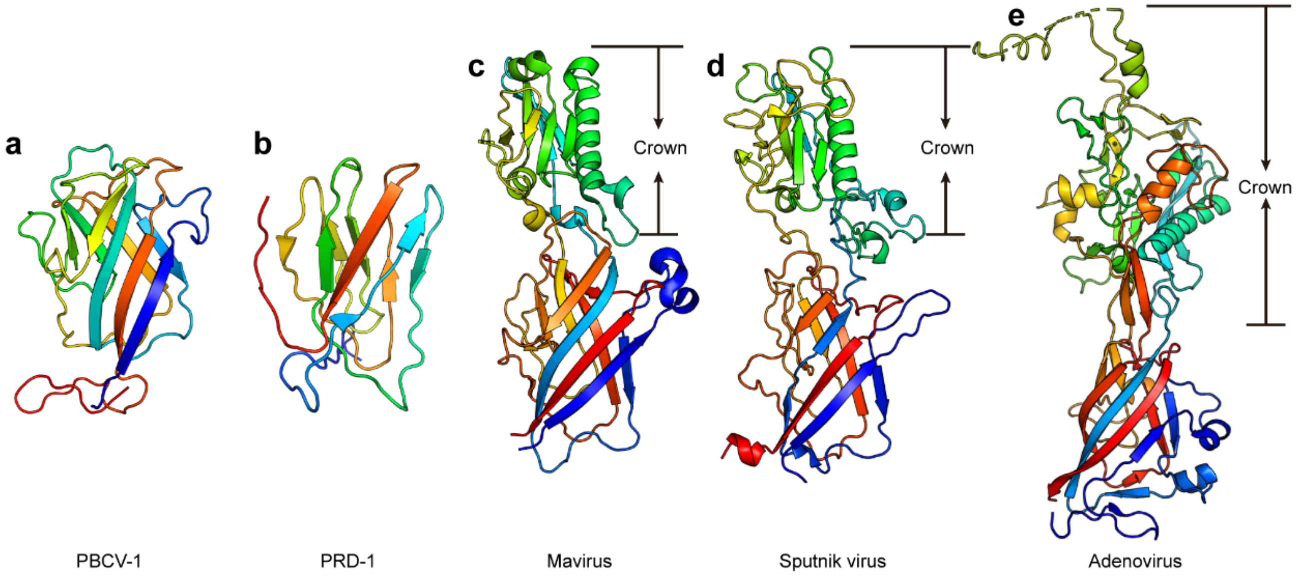

Supplementary Fig. 4 | Structural comparison of penton proteins with a single JR fold. The atomic models of the penton proteins in PBCV-1 (PDB: 6NCL)[2] (**a**), PRD-1 (PDB: 1W8X)[16] (**b**), mavirus (PDB: 6G42)[6] (**c**), sputnik virus (PDB: 3J26)[8] (**d**), and AdV (PDB: 1X9P)[17] (**e**) are shown as ribbons. Rainbow coloring from blue to red indicates the N- to C-terminus of the residues in each model.

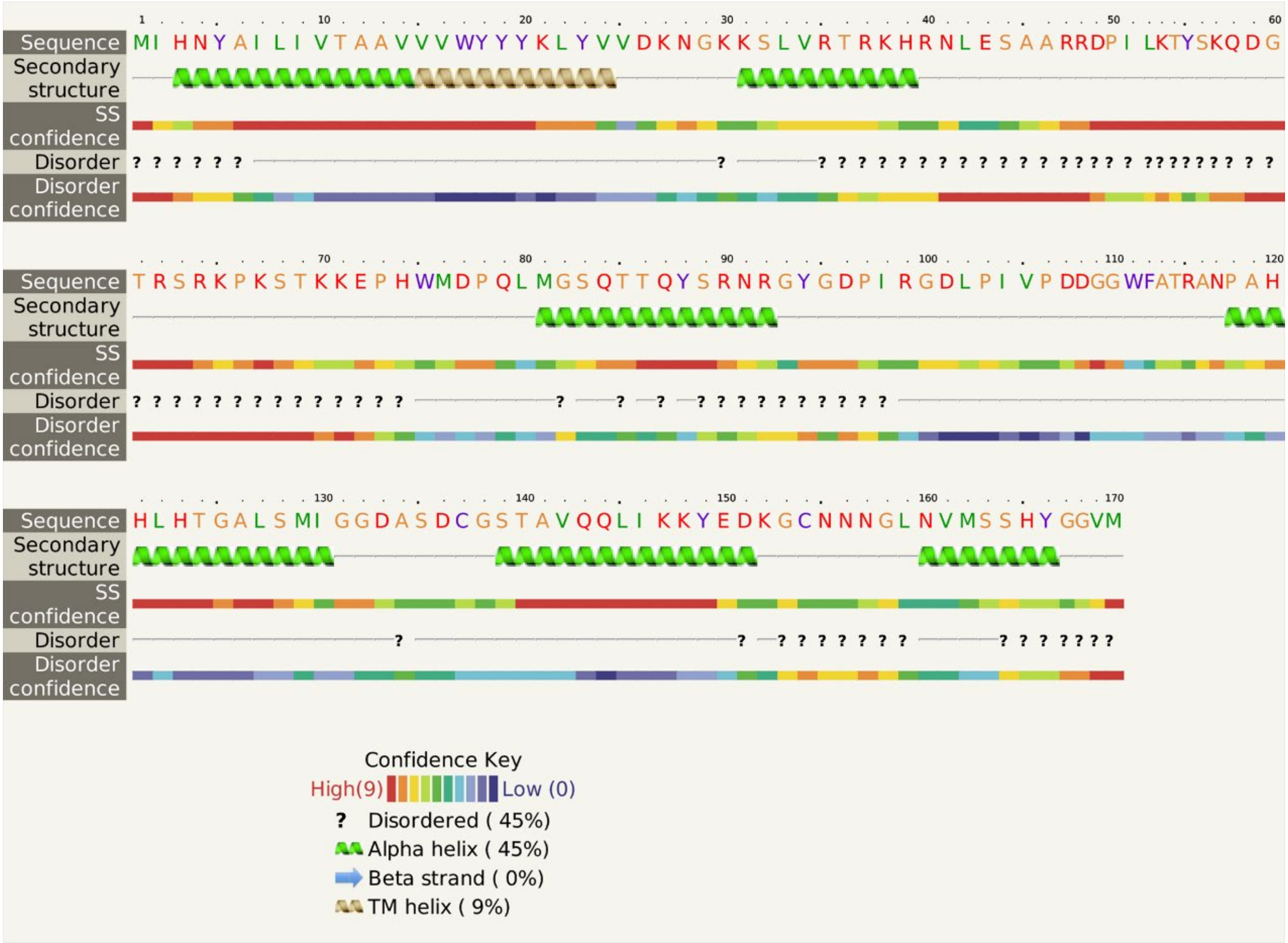

Supplementary Fig. 5 | Secondary structure prediction of VP38. The PHYRE2 Protein Fold Recognition Server[15] was used to predict the secondary structure of VP38.

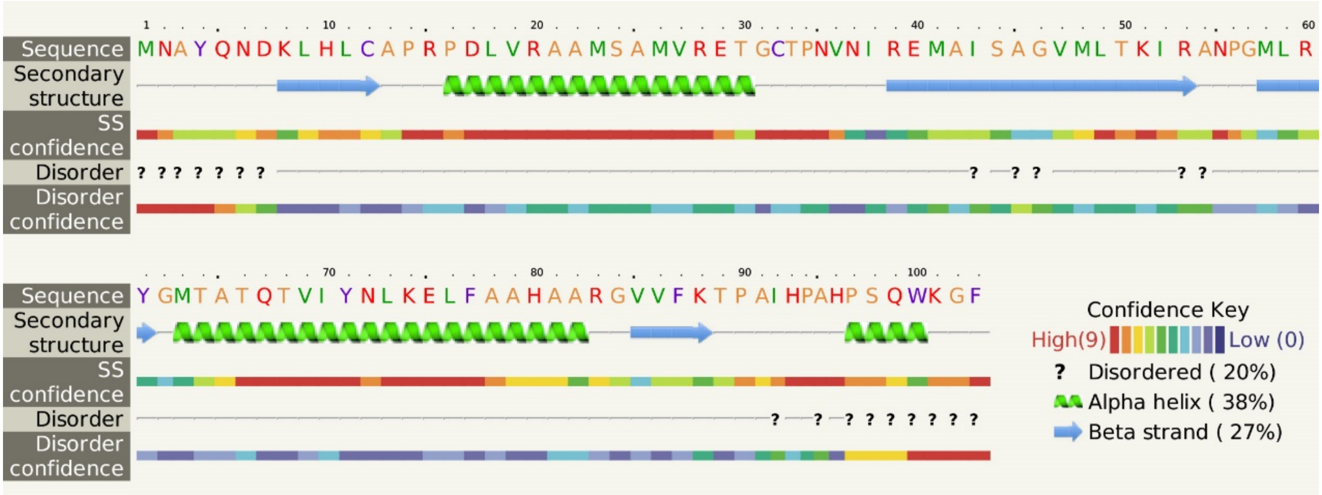

Supplementary Fig. 6 | Secondary structure prediction of VP139. The PHYRE2 Protein Fold Recognition Server[15] was used to predict the secondary structure of VP139.

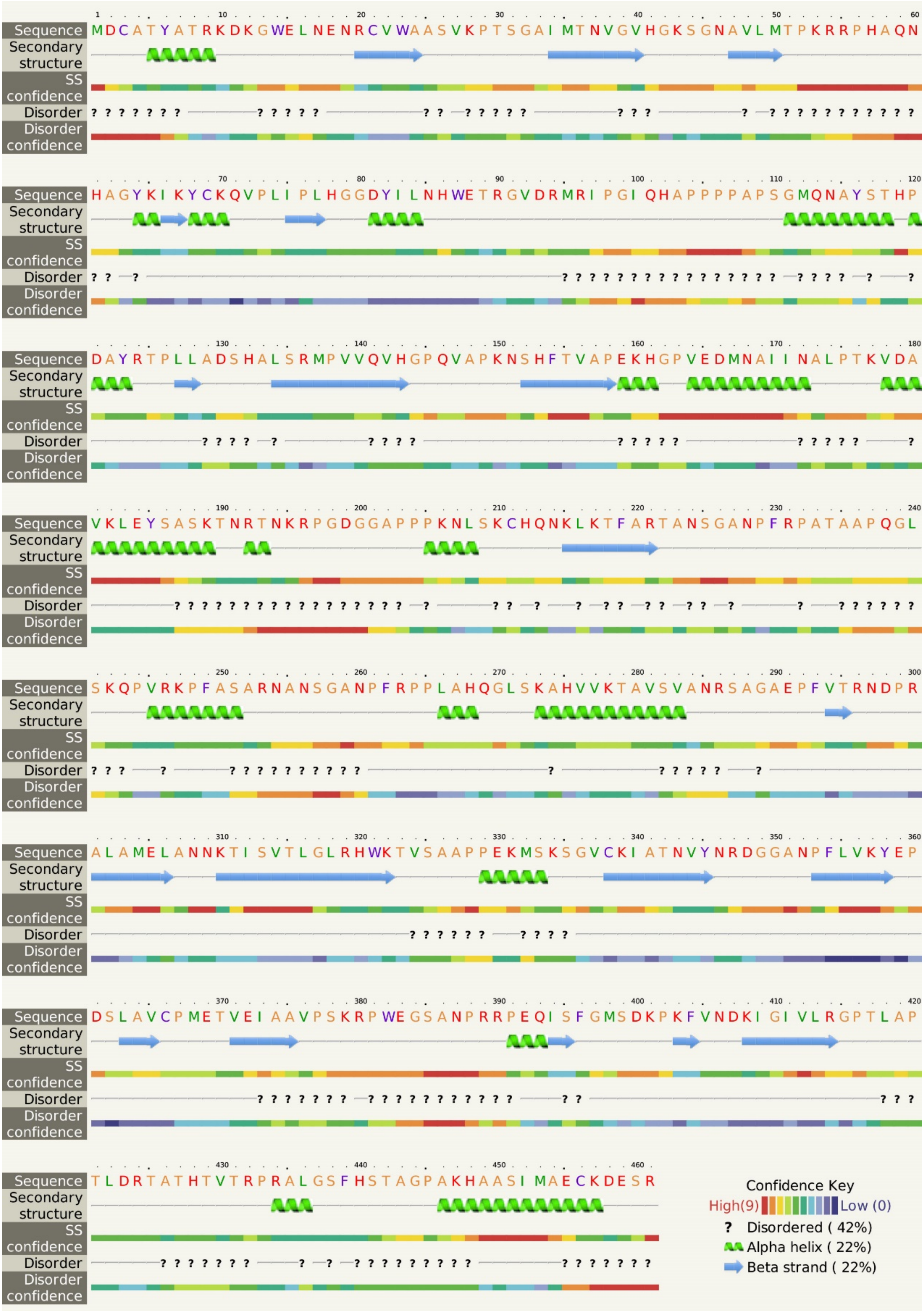
Sequence
Secondary structure
SS confidence
Disorder
Disorder confidence
MDCATYATRKDKGWELNENRCVWAASVKPTSGAIMTNVGVHGKSGNAVLMTPKRRPHAQN
HAGYKIKYCKQVPLIPLHGGDYILNHWETRGVDRMRIPGIQHAPPPPAPSGMQNAYSTHP
DAYRTPLLADSHALSRMPVVQVHGPQVAPKNSHFTVAPEKHGPVEDMNAIINALPTKVDA
VKLEYSASKTNRTNKRPGDGGAPPPKNLSKCHQNKLKTFARTANSGANPFRPATAAPQGL
SKQPVRKPFASARNANSGANPFRPPLAHQGLSKAHVVKTAVSVANRSAGAEPFVTRNDPR
ALAMELANNKTISVTLGLRHWKTVSAAPPEKMSKSGVCKIATNVYNRDGGANPFLVKYEP
DSLAVCPMETVEIAAVPSKRPWEGSANPRRPEQISFGMSDKPKFVNDKIGIVLRGPTLAP
TLDRTATHTVTRPRALGSFHSTAGPAKHAASIMAECKDESR
Confidence Key
High(9)
Low (0)
? Disordered (42%)
Alpha helix (22%)
Beta strand (22%)

Supplementary Fig. 7 | Secondary structure prediction of VP137. The PHYRE2 Protein Fold Recognition Server[15] was used to predict the secondary structure of VP137.

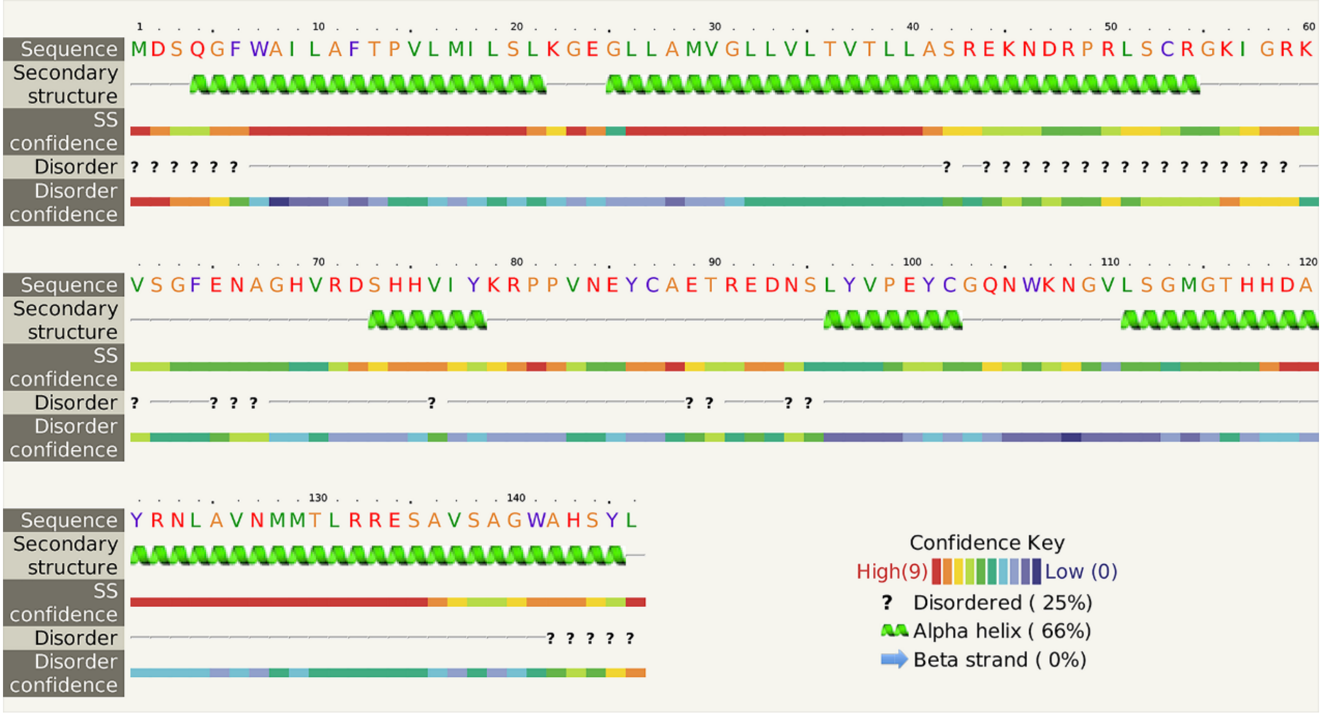

Supplementary Fig. 8 | Secondary structure prediction of VP59. The PHYRE2 Protein Fold Recognition Server[15] was used to predict the secondary structure of VP59.

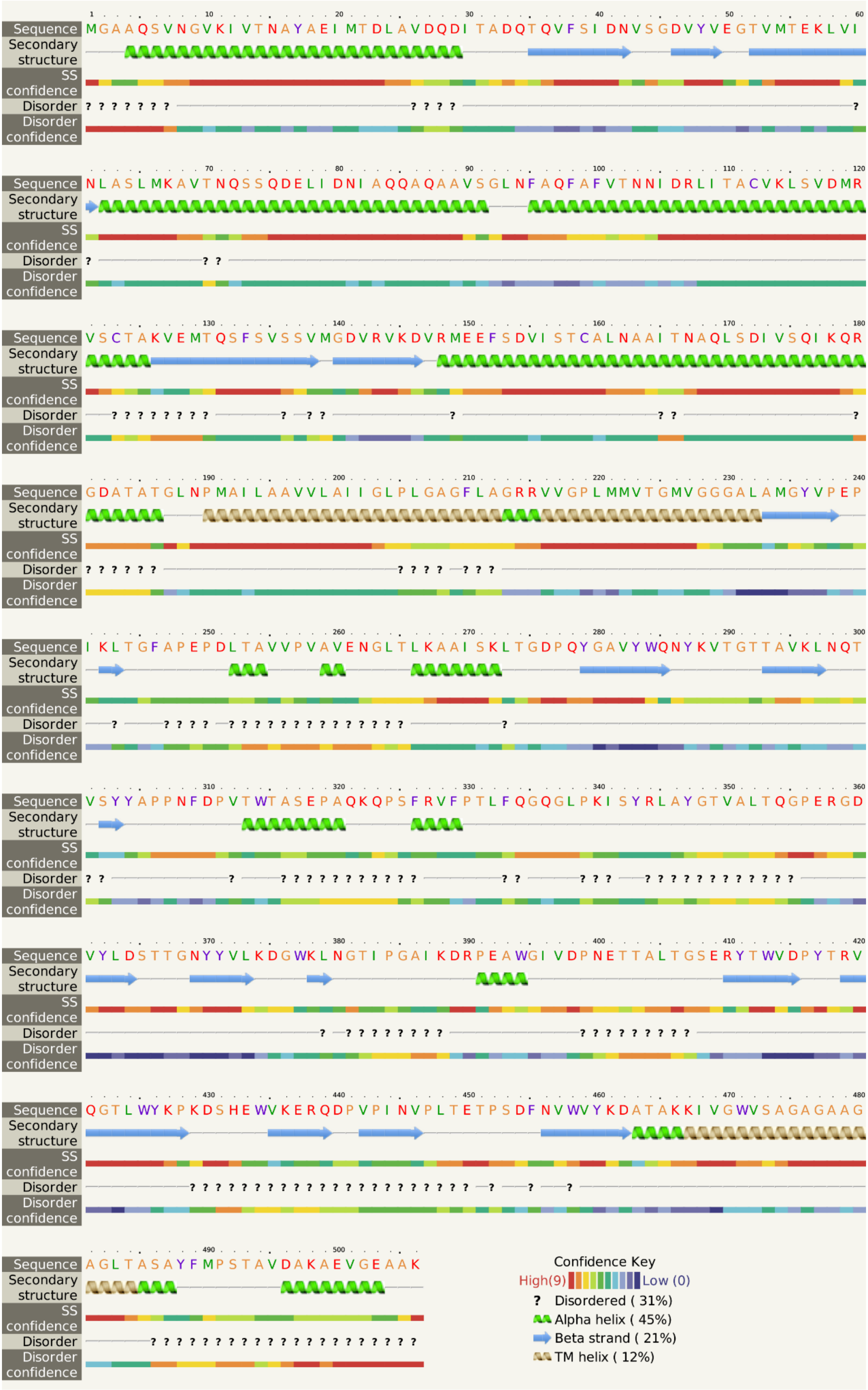
Sequence
Secondary structure
SS confidence
Disorder
Disorder confidence
Confidence Key
High(9) Low (0)
? Disordered (31%)
Alpha helix (45%)
Beta strand (21%)
TM helix (12%)

Supplementary Fig. 9 | Secondary structure prediction of VP88. The PHYRE2 Protein Fold Recognition Server[15] was used to predict the secondary structure of VP88.

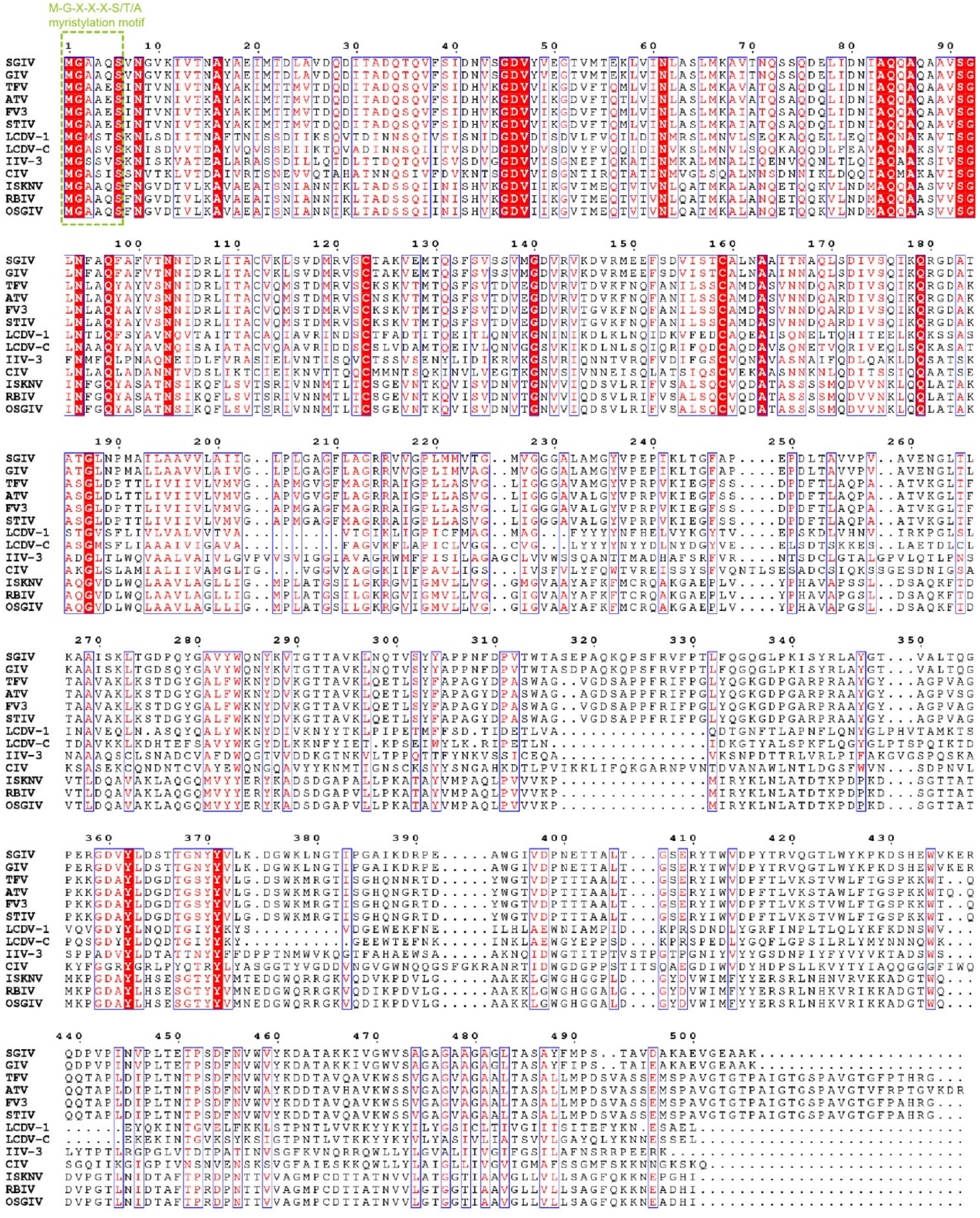

Supplementary Fig. 10 | AA sequence alignment of the anchor proteins in iridovirids. The VP88 orthologs were identified from 13 iridovirid genomes using the BLAST[18,19]. The AA sequence alignment was generated by ClustalW and ESPript 3.0[20]. Conserved residues are highlighted in red. The myristylation sites are outlined

in green dashed lines. GenBank accession codes are listed as follows: SGIV, AY521625; Grouper iridovirus (GIV), AY666015; Tiger frog virus (TFV), AF389451; Ambystoma tigrinum stebbensi virus (ATV), AY150217; FV3, AY548484; Soft-shelled turtle iridovirus (STIV), EU627010; Lymphocystis disease virus 1 (LCDV-1), NC_001824; Lymphocystis disease virus-isolate China (LCDV-C), AY380826; Invertebrate iridescent virus 3 (IIV-3), DQ643392; CIV, AF303741; Infectious spleen and kidney necrosis virus (ISKNV), AF371960; Rock bream iridovirus (RBIV), AY532606; and Orange-spotted grouper iridovirus (OSGIV), AY894343.

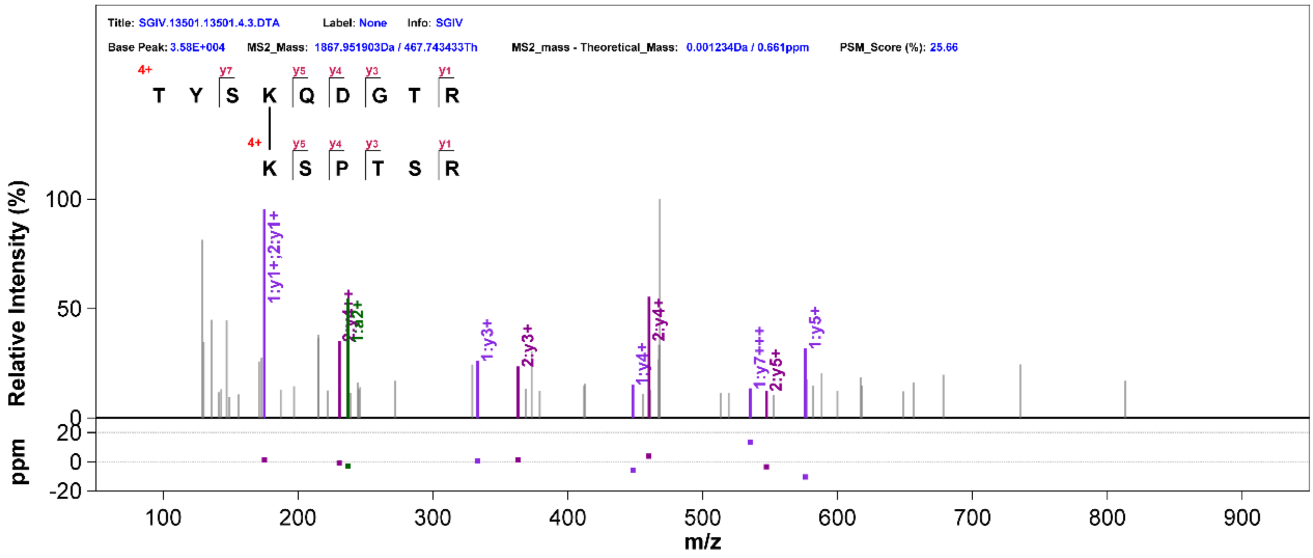

Supplementary Fig. 11 | Crosslinking between VP22 and VP38. The purified SGIV sample was crosslinked with BS^3. Crosslinking between residue K47 of VP22 and residue K57 of VP38 was identified. Raw CXMS data were deposited into the ProteomeXchange Consortium.

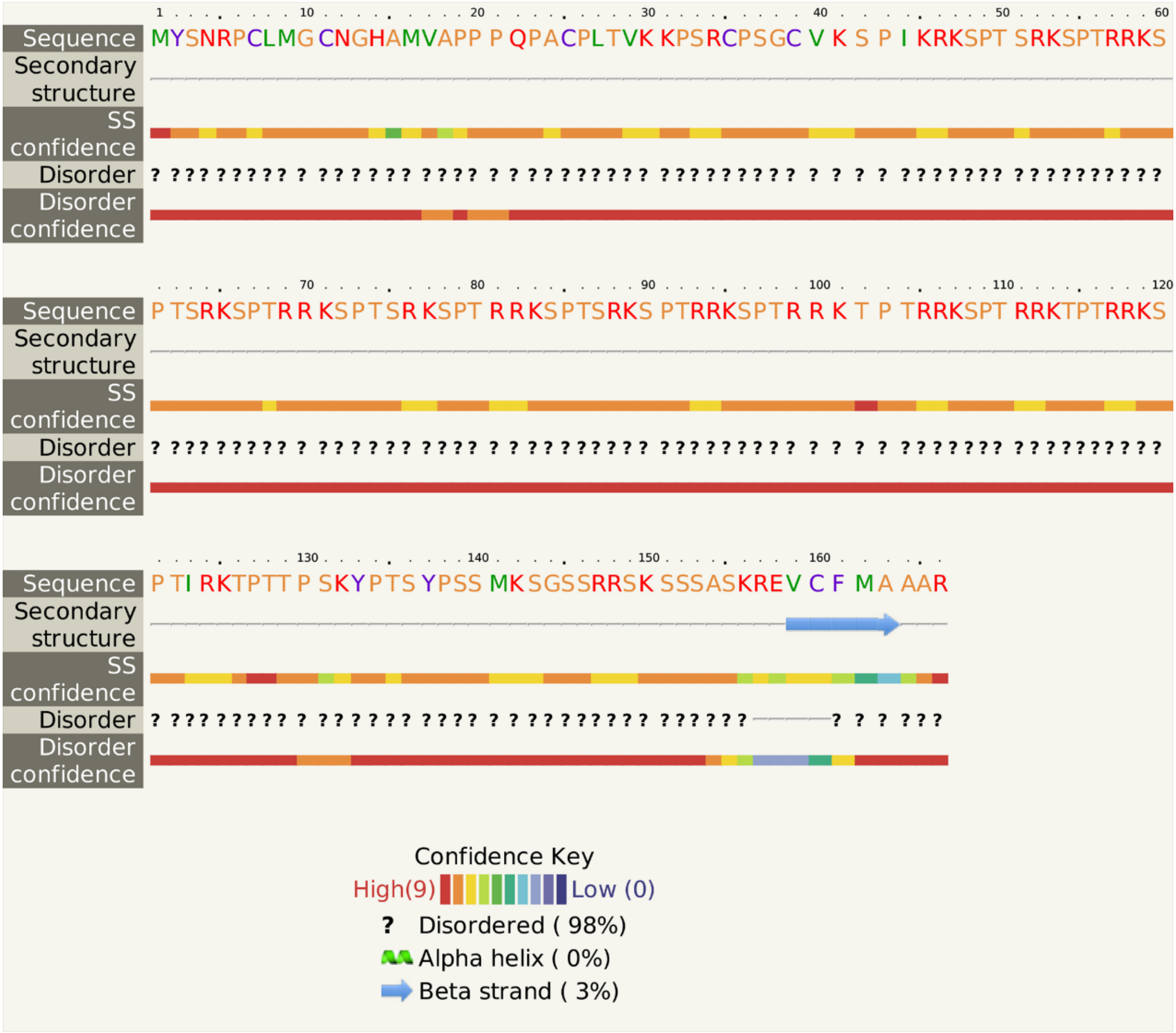

Supplementary Fig. 12 | Secondary structure prediction of VP22. The PHYRE2 Protein Fold Recognition Server[15] was used to predict the secondary structure of VP22.

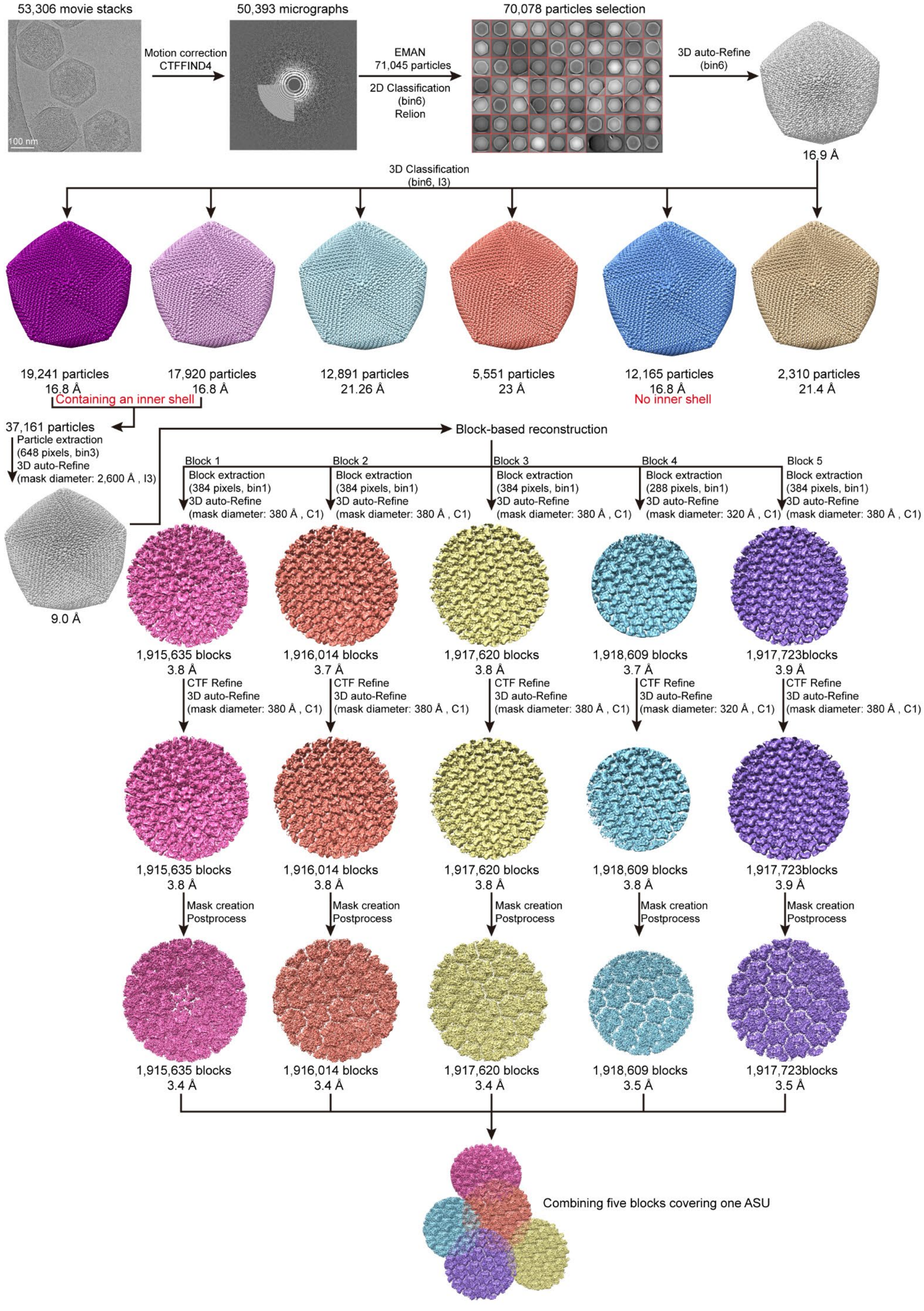
53,306 movie stacks
100 nm
Motion correction
CTFFIND4
50,393 micrographs
EMAN
71,045 particles
2D Classification
(bin6)
Relion
70,078 particles selection
3D auto-Refine
(bin6)
16.9 Å
3D Classification
(bin6, I3)
19,241 particles
16.8 Å
17,920 particles
16.8 Å
12,891 particles
21.26 Å
5,551 particles
23 Å
12,165 particles
16.8 Å
2,310 particles
21.4 Å
Containing an inner shell
No inner shell
37,161 particles
Particle extraction
(648 pixels, bin3)
3D auto-Refine
(mask diameter: 2,600 Å , I3)
9.0 Å
Block-based reconstruction
Block 1
Block extraction
(384 pixels, bin1)
3D auto-Refine
(mask diameter: 380 Å , C1)
Block 2
Block extraction
(384 pixels, bin1)
3D auto-Refine
(mask diameter: 380 Å , C1)
Block 3
Block extraction
(384 pixels, bin1)
3D auto-Refine
(mask diameter: 380 Å , C1)
Block 4
Block extraction
(288 pixels, bin1)
3D auto-Refine
(mask diameter: 320 Å , C1)
Block 5
Block extraction
(384 pixels, bin1)
3D auto-Refine
(mask diameter: 380 Å , C1)
1,915,635 blocks
3.8 Å
1,916,014 blocks
3.7 Å
1,917,620 blocks
3.8 Å
1,918,609 blocks
3.7 Å
1,917,723blocks
3.9 Å
CTF Refine
3D auto-Refine
(mask diameter: 380 Å , C1)
CTF Refine
3D auto-Refine
(mask diameter: 380 Å , C1)
CTF Refine
3D auto-Refine
(mask diameter: 380 Å , C1)
CTF Refine
3D auto-Refine
(mask diameter: 320 Å , C1)
CTF Refine
3D auto-Refine
(mask diameter: 380 Å , C1)
1,915,635 blocks
3.8 Å
1,916,014 blocks
3.8 Å
1,917,620 blocks
3.8 Å
1,918,609 blocks
3.8 Å
1,917,723blocks
3.9 Å
Mask creation
Postprocess
Mask creation
Postprocess
Mask creation
Postprocess
Mask creation
Postprocess
Mask creation
Postprocess
1,915,635 blocks
3.4 Å
1,916,014 blocks
3.4 Å
1,917,620 blocks
3.4 Å
1,918,609 blocks
3.5 Å
1,917,723blocks
3.5 Å
Combining five blocks covering one ASU

Supplementary Fig. 13 | Flowchart for cryo-EM data processing. Data processing and block-based reconstruction of the SGIV capsid. Details can be found in Methods.

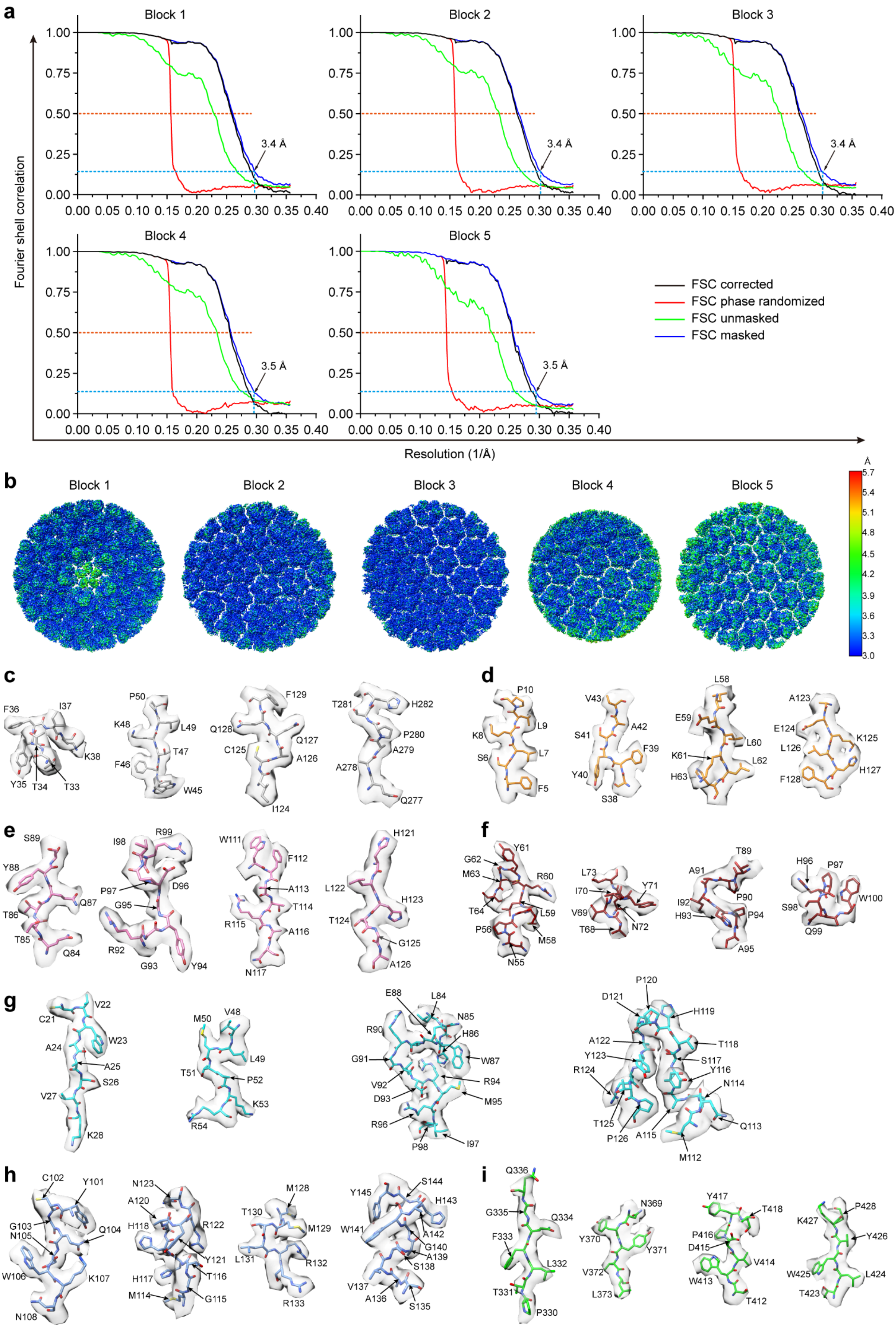

a
Block 1
Block 2
Block 3
Block 4
Block 5
Fourier shell correlation
Resolution (1/Å)
3.4 Å
3.4 Å
3.4 Å
3.5 Å
3.5 Å
FSC corrected
FSC phase randomized
FSC unmasked
FSC masked
b
Block 1
Block 2
Block 3
Block 4
Block 5
Å
c
d
e
f
g
h
i

Supplementary Fig. 14 | Resolution estimation for five blocks and cryo-EM maps of MCP and mCPs.

a FSC curves of the five blocks. The resolutions are shown based on the FSC=0.143 criterion. **b** Local resolution estimation of the five blocks using Resmap[21]. **c-i** Representative densities around the MCP (**c**), penton protein (**d**), VP38 (**e**), VP139 (**f**), VP137 (**g**), VP59 (**h**), and VP88 (**i**).

Supplementary Table 1. Viral proteins identified by proteomics of the purified SGIV sample.

Gene	Description	Coverage [%]	No. of unique peptides	No. of amino acids	Molecular weight [kDa]
ORF001L	Uncharacterized protein	6	2	304	34.6
ORF003R	3-beta-hydroxy-delta-5-C27-steroid oxidoreductase	13	5	381	43.1
ORF004L	Uncharacterized protein	17	6	365	41.6
ORF006R	Uncharacterized protein	36	11	259	29.1
ORF007L	Uncharacterized protein	8	2	307	30.1
ORF008L	Uncharacterized protein	65	11	230	22.1
ORF009L	Uncharacterized protein	18	3	154	16.4
ORF011L	Uncharacterized protein	19	1	62	6.6
ORF012L	Rho_N domain-containing protein	50	59	1024	117.3
ORF014L	Uncharacterized protein	34	5	141	15.8
ORF016L	Uncharacterized protein	44	14	413	46.2
ORF018R	Uncharacterized protein	67	16	285	32.3
ORF019R	Uncharacterized protein	32	9	342	36.8
ORF020L	Uncharacterized protein	34	7	322	35.4
ORF021L	Uncharacterized protein	58	9	139	16.4
ORF022L	Uncharacterized protein	24	7	166	18.6
ORF025L	SAP domain-containing protein	19	8	510	56.5
ORF026R	Uncharacterized protein	57	23	566	63.3
ORF029L	Uncharacterized protein	8	3	332	36.6
ORF035L	Uncharacterized protein	3	1	375	42.2
ORF036L	Uncharacterized protein	14	5	329	37.3
ORF038L	Uncharacterized protein	65	8	170	19
ORF039L	Protein kinase domain-containing protein	57	63	1051	118.2
ORF043R	Uncharacterized protein	50	31	667	73.6
ORF045L	Uncharacterized protein	40	7	242	22.9
ORF046L	Uncharacterized protein	46	6	248	23.8
ORF047L	Ribonucleoside-diphosphate reductase beta subunit	6	3	384	43.6
ORF049L	dUTPase	49	6	155	17
ORF052L	D5 family NTPase	13	10	968	109.8
ORF055R	Uncharacterized protein	82	17	240	22.8
ORF056R	Uncharacterized protein	46	8	246	22.9
ORF057L	Uncharacterized protein	32	32	1168	131.2
ORF059L	Uncharacterized protein	59	8	146	16.3
ORF060R	NTPase	46	38	970	109.6
ORF061R	FCP1 homology domain-containing protein	48	9	204	23.2
ORF064R	Ribonucleoside-diphosphate reductase alpha subunit	19	9	572	63.7
ORF067L	Deoxynucleoside kinase	72	14	191	21.6
ORF068L	Uncharacterized protein	10	2	272	29.6
ORF069L	Uncharacterized protein	63	35	548	61.8
ORF070R	Sulfhydryl oxidase	53	7	152	17
ORF072R	Major capsid protein	81	24	463	50.5
ORF073L	DNA-directed RNA polymerase subunit beta	3	3	1103	123.3
ORF075R	Uncharacterized protein	65	14	178	19.9
ORF078L	Tyrosine kinase	46	32	790	88.1
ORF081L	Tyrosine kinase	33	8	187	21.8

ORF082L	Uncharacterized protein	24	5	222	24.3
ORF083R	Uncharacterized protein	11	4	445	50.4
ORF084L	RNase III	48	21	375	41.6
ORF086R	Putative immediate-early protein	78	14	154	17.1
ORF088L	Uncharacterized protein	32	12	506	54
ORF089L	Uncharacterized protein	41	19	390	45.6
ORF090L	Uncharacterized protein	65	20	373	43.5
ORF091L	Uncharacterized protein	10	3	378	44.5
ORF093L	Uncharacterized protein	44	18	405	47.6
ORF098R	Uncharacterized protein	23	4	267	30.5
ORF101R	Uncharacterized protein	67	19	313	35
ORF102L	Ubiqutin/ribosomal protein	77	6	77	8.5
ORF103R	Uncharacterized protein	20	2	97	11
ORF111R	Uncharacterized protein	20	5	255	29.2
ORF112R	Uncharacterized protein	5	1	355	34.6
ORF114L	Uncharacterized protein	9	1	112	12
ORF115R	Uncharacterized protein	14	1	152	17.2
ORF118R	Uncharacterized protein	13	5	319	35.6
ORF119R	Uncharacterized protein	75	6	83	9.1
ORF121R	Uncharacterized protein	19	1	84	9.8
ORF122L	Uncharacterized protein	30	7	210	24.2
ORF123L	Uncharacterized protein	10	4	362	41.5
ORF125R	Uncharacterized protein	14	2	184	21.1
ORF128R	DNA polymerase	15	14	1009	114.9
ORF131R	Ig-like domain-containing protein	11	2	184	19.9
ORF132R	Uncharacterized protein	4	1	275	31.3
ORF134L	ATPase	45	12	323	36.5
ORF135L	Uncharacterized protein	19	2	112	12.9
ORF136R	LITAF domain-containing protein	50	4	104	11.6
ORF137R	Uncharacterized protein	59	25	461	49.7
ORF139R	Uncharacterized protein	55	5	103	11.3
ORF140R	Uncharacterized protein	24	7	304	32.1
ORF143L	Uncharacterized protein	20	1	79	8.9
ORF146L	NTPase/helicase	44	13	324	36.7
ORF147L	Uncharacterized protein	3	1	344	39.4
ORF149R	Uncharacterized protein	12	2	125	14.6
ORF150L	Phosphotransferase	9	5	508	57.2
ORF151L	Uncharacterized protein	35	6	195	22.3
ORF152R	Helicase	32	12	412	46.5
ORF155R	Sema domain-containing protein	17	10	575	64.6
ORF156L	Uncharacterized protein	58	21	270	31.1
ORF158L	Uncharacterized protein	13	2	138	15.8
ORF159R	Uncharacterized protein	8	1	163	17.4
ORF162L	Uncharacterized protein	57	19	382	44.1

Supplementary Table 2. Statistics for Cryo-EM imaging, data processing, and model refinement.

Data collection							
Microscope	FEI Titan Krios						
Camera	Falcon3						
Magnification	59,000x						
Voltage (kV)	300						
Total dose (e^{-}/Å^{2})	50						
Exposure time (s)	1						
Number of frames	39						
Defocus range (μm)	1.3 to 1.8						
Pixel size (Å)	1.4						
Data processing							
	Block 1 (EMD-34227)	Block 2 (EMD-34230)	Block 3 (EMD-34235)	Block 4 (EMD-34229)	Block 5 (EMD-34236)	Composite map for five blocks (EMD-34815)	Intact virion (EMD-34251)
Symmetry imposed	C1	C1	C1	C1	C1	C1	I3
Final blocks/particles images	1,915,635	1,916,014	1,917,620	1,918,609	1,917,723	-	37,161
Map resolution (Å)	3.4	3.4	3.4	3.5	3.5	3.5	9.0
Model refinement for one ASU (PDB: 8HIF)							
Model composition							
Non-hydrogen atoms	450,820						
Protein residues	59,078						
Ligands	0						
Validation							
MolProbity score	1.75						
Clash score	7.72						
Poor rotamers (%)	0.07						
R.m.s. deviations							
Bond lengths (Å)	0.002						
Bond angles (°)	0.508						
Ramachandran plot							
Favored (%)	95.23						
Allowed (%)	4.70						
Disallowed (%)	0.07						

Supplementary References

1. Yan, X. *et al.* The capsid proteins of a large, icosahedral dsDNA virus. *J. Mol. Biol.* **385,** 1287–1299 (2009).
2. Fang, Q. *et al.* Near-atomic structure of a giant virus. *Nat. Commun.* **10,** 388 (2019).
3. Klose, T. *et al.* Structure of faustovirus, a large dsDNA virus. *Proc. Natl. Acad. Sci. U. S. A.* **113,** 6206–6211 (2016).
4. Wang, N. *et al.* Architecture of African swine fever virus and implications for viral assembly. *Science* **366,** 640–644 (2019).
5. Xiao, C. *et al.* Cryo-EM reconstruction of the Cafeteria roenbergensis virus capsid suggests novel assembly pathway for giant viruses. *Sci. Rep.* **7,** 5484 (2017).
6. Born, D. *et al.* Capsid protein structure, self-assembly, and processing reveal morphogenesis of the marine virophage mavirus. *Proc. Natl. Acad. Sci. U. S. A.* **115,** 7332–7337 (2018).
7. Castro, C. de *et al.* Structure of the chlorovirus PBCV-1 major capsid glycoprotein determined by combining crystallographic and carbohydrate molecular modeling approaches. *Proc. Natl. Acad. Sci. U. S. A.* **115,** E44-E52 (2018).
8. Zhang, X. *et al.* Structure of Sputnik, a virophage, at 3.5-Å resolution. *Proc. Natl. Acad. Sci. U. S. A.* **109,** 18431–18436 (2012).
9. Laanto, E. *et al.* Virus found in a boreal lake links ssDNA and dsDNA viruses. *Proc. Natl. Acad. Sci. U. S. A.* **114,** 8378–8383 (2017).
10. Abrescia, N. G. A. *et al.* Insights into virus evolution and membrane biogenesis from the structure of the marine lipid-containing bacteriophage PM2. *Mol. Cell* **31,** 749–761 (2008).
11. Benson, S. D., Bamford, J. K., Bamford, D. H. & Burnett, R. M. Viral Evolution Revealed by Bacteriophage PRD1 and Human Adenovirus Coat Protein Structures. *Cell* **98,** 825–833 (1999).
12. Khayat, R. *et al.* Structure of an archaeal virus capsid protein reveals a common ancestry to eukaryotic and bacterial viruses. *Proc. Natl. Acad. Sci. U. S. A.* **102,** 18944–18949 (2005).
13. Bahar, M. W., Graham, S. C., Stuart, D. I. & Grimes, J. M. Insights into the evolution of a complex virus from the crystal structure of vaccinia virus D13. *Structure* **19,** 1011–1020 (2011).
14. Dai, X., Wu, L., Sun, R. & Zhou, Z. H. Atomic Structures of Minor Proteins VI and VII in Human Adenovirus. *J. Virol.* **91** (2017).
15. Kelley, L. A., Mezulis, S., Yates, C. M., Wass, M. N. & Sternberg, M. J. E. The Phyre2 web portal for protein modeling, prediction and analysis. *Nat. Protoc.* **10,** 845–858 (2015).
16. Abrescia, N. G. A. *et al.* Insights into assembly from structural analysis of bacteriophage PRD1. *Nature* **432,** 68–74 (2004).
17. Zubieta, C., Schoehn, G., Chroboczek, J. & Cusack, S. The structure of the human adenovirus 2 penton. *Mol. Cell* **17,** 121–135 (2005).
18. Altschul, S. F., Gish, W., Miller, W., Myers, E. W. & Lipman, D. J. Basic local alignment search tool. *J. Mol. Biol.* **215,** 403–410 (1990).
19. Mount, D. W. Using the Basic Local Alignment Search Tool (BLAST). *CSH Protoc.* **2007,** pdb.top17 (2007).
20. Robert, X. & Gouet, P. Deciphering key features in protein structures with the new ENDscript server. *Nucleic Acids Res.* **42,** 320-324 (2014).
21. Kucukelbir, A., Sigworth, F. J. & Tagare, H. D. Quantifying the local resolution of cryo-EM density maps. *Nat. Methods*

11, 63–65 (2014).
